# TGF-α/EGFR-mediated lymphatic metastasis reveals a repositionable therapeutic target in breast cancer

**DOI:** 10.1038/s41523-026-00941-0

**Published:** 2026-04-03

**Authors:** Wenyang Shi, Yueyun Pan, Bhavik Rathod, Yuhan Wang, Zhi Wang, Jianyu Shen, Francesca Gatto, Mingzhi Liu, Yizhe Sun, Margareta Wilhelm, Thomas Helleday, Maria H. Ulvmar, Sara H. Windahl, Mikael C. I. Karlsson, Jonas Fuxe

**Affiliations:** 1 Division of Pathology, Department of Laboratory Medicine, Karolinska Institutet, Huddinge, Sweden; 2 Department of Microbiology, Tumor and Cell Biology, Karolinska Institutet, Stockholm, Sweden; 3 Department of Medical Biochemistry and Microbiology, Uppsala University, Uppsala, Sweden; 4 Science for Life Laboratory, Department of Oncology-Pathology, Karolinska Institutet, Solna, Sweden

**Keywords:** Cancer, Immunology, Oncology

## Abstract

The epidermal growth factor receptor (EGFR) is a well-established oncogenic driver in multiple epithelial cancers, yet its role in breast cancer remains elusive, with EGFR-targeted therapies showing limited clinical efficacy. Here, we demonstrate that EGFR promotes selective lymphatic dissemination in triple-negative breast cancer through a chemotactic mechanism involving the EGFR ligand TGF-α. Lymphatic endothelial cells (LECs) were identified as a tumor-associated source of TGF-α, both in a murine model and in human breast cancer, particularly upon stimulation with TGF-β1, a cytokine commonly overexpressed in breast tumors associated with lymph metastasis. We found that TGF-α–EGFR interactions elicit directional migration via STAT3 signaling, whereas the co-secreted ligand CTGF, enriched in blood endothelial cells, suppressed migration. Pharmacologic blockade of TGF-α with Fepixnebart, a first-in-class ligand-neutralizing antibody targeting TGF-α and previously not tested in oncologic indications, significantly inhibited early lymph metastasis of EGFR⁺ tumor cells. Furthermore, EGFR overexpression resulted in increased cellularity in tumor-draining lymph nodes and reduced CD8⁺ T-cell representation. Together, these findings reveal a role for the TGF-α/EGFR axis in lymph metastasis and propose a rationale for repositioning EGFR-targeted therapies toward targeting early metastatic spread and immunomodulation in breast cancer.

## Introduction

EGFR is implicated in multiple stages of tumor progression, including proliferation, survival, invasion, and metastasis of many epithelial cancers. EGFR-targeted therapies have demonstrated clinical efficacy in several epithelial cancers, including non-small cell lung cancer (NSCLC) and colorectal carcinoma, where EGFR is frequently activated by mutation or gene amplification and serves as a driver of tumor growth^1–3^. In these contexts, EGFR tyrosine kinase inhibitors (TKIs) and monoclonal antibodies have transformed treatment paradigms and significantly improved patient outcomes.

In breast cancer, however, the role of EGFR is less clear. EGFR is not commonly mutated, yet it is frequently overexpressed, particularly in the basal-like and triple-negative breast cancer (TNBC) subtypes^4,5^. Elevated EGFR expression in these subtypes correlates with poor prognosis, early lymph node involvement, and resistance to therapy^2,6^. However, multiple clinical trials evaluating EGFR inhibitors in breast cancer have yielded disappointing results, with limited or no improvement in progression-free survival^4,5,7^. These outcomes have often been attributed to innate or acquired resistance, lack of EGFR addiction, and subtype-specific signaling redundancy^8^.

This disconnect between EGFR expression and therapeutic efficacy suggests that EGFR may exert non-canonical functions in breast cancer that are not adequately captured by traditional cytostatic response measures. Recent evidence has indicated that EGFR may modulate the tumor microenvironment, facilitate epithelial-to-mesenchymal transition (EMT), and contribute to immune evasion in inflammatory breast cancer^9^. It has also been reported that circulating tumor cells in the lymphatic compartment express higher levels of EGFR than those in the bloodstream, implicating a possible role for EGFR in lymphatic dissemination^10^.

Lymphatic spread is a critical step in breast cancer progression and is associated with poor clinical outcome and immunosuppression^11–13^. Although the lymphatic system is often viewed as a passive conduit for tumor cells, recent findings suggest a more active role for lymphatic endothelial cells (LECs) in shaping the metastatic niche^14^. LECs can produce chemokines that attract tumor cells and facilitate pre-metastatic niche formation^14^. The cytokine transforming growth factor beta (TGF-β), an inducer of EMT, immune evasion, and metastasis^15^, is frequently overexpressed in breast cancer and correlates with lymph node involvement^16^. Recent data show that TGF-β can activate LECs to produce chemotactic factors that facilitate directional tumor cell migration^14,17,18^, suggesting a role in promoting crosstalk between tumor cells and their lymphatic microenvironment.

In this study, we demonstrate that EGFR drives lymphatic metastasis, without altering tumor growth or hematogenous dissemination, in a syngeneic mouse model of TNBC. We identify LECs as a source of EGFR ligands, particularly TGF-α, which was upregulated and secreted by TGF-β1-activated LECs. Using chemotaxis assays and targeted inhibition, we found that TGF-α is both necessary and sufficient for EGFR-mediated migration and lymphatic invasion. Mechanistically, this response was dependent on STAT3 activation, and therapeutic blockade of TGF-α using the first-in-class antibody Fepixnebart disrupted lymphatic metastasis. Finally, we show that EGFR overexpression accelerates early immunosuppression in tumor-draining lymph nodes, indicating a dual role in both physical dissemination and immune escape. Together, our findings uncover a novel, growth-independent function of EGFR in lymphatic metastasis and highlight the therapeutic potential of repositioning EGFR-targeting agents to prevent early dissemination in breast cancer.

## Results

### EGFR overexpression promotes lymphatic metastasis without affecting tumor growth or distant spread

EGFR is frequently overexpressed in basal-like, triple-negative breast cancer (TNBC), and its expression has been linked to lymph node metastasis in clinical studies. However, despite its oncogenic potential, EGFR-targeted therapies have demonstrated limited efficacy in breast cancer, suggesting that EGFR may contribute to disease progression through mechanisms beyond tumor cell proliferation. To explore this, we analyzed EGFR expression in breast cancer cell lines. EGFR was highly expressed in the human TNBC cell line MDA-MB-231, which displays a basal-like phenotype, but low in MCF-7 cells, which displays a luminal phenotype (Supplementary Fig. S1a). Although a commonly used TNBC model, the expression of EGFR was low in murine 4T1 cells. 4T1 cells are a well-established model for spontaneous metastasis—predominantly to lung and bone, but disseminates infrequently via the lymphatic system. Treatment of 4T1 and MDA-MB-231 cells with recombinant TGF-β1 upregulated EGFR levels (Supplementary Fig. S1b, c), supporting a role for TGF-β1 in priming breast cancer cells for EGFR-dependent responses in the metastatic niche.

To develop an immunocompetent in vivo model for functional studies, we stably transduced 4T1 cells with a murine EGFR expression construct, generating 4T1-EGFR cells. EGFR overexpression was confirmed by qPCR, western blot, and immunofluorescence (Supplementary Fig. S1d–f). No differences were found between the 4T1 vector and 4T1-EGFR cells in terms of morphology (Supplementary Fig. S1g) or proliferation (Supplementary Fig. S1h).

**Fig. 1:**
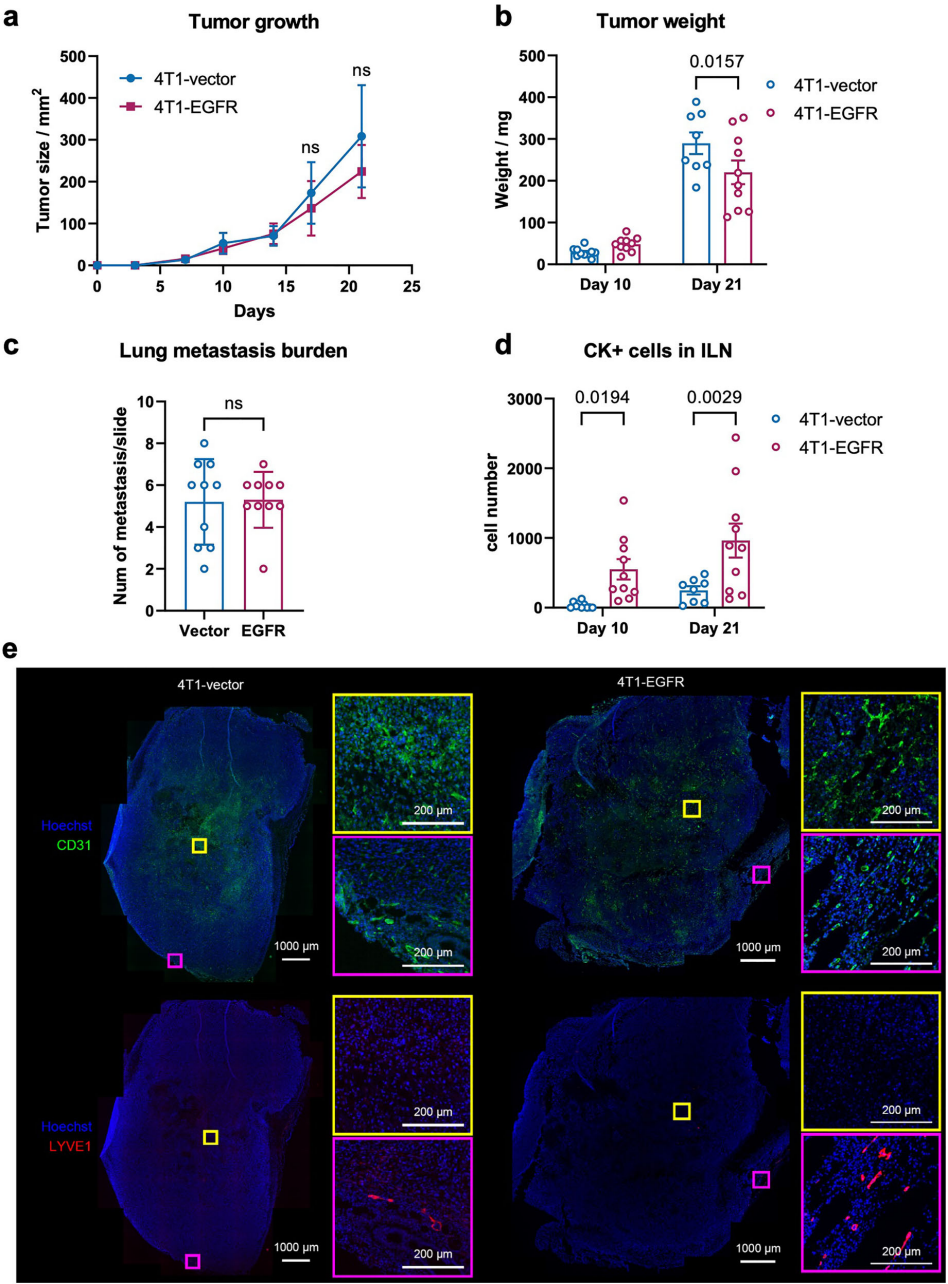
EGFR promotes lymph node metastasis independent of tumor growth or distant spread. **a** Tumor growth of 4T1-vector and 4T1-EGFR cells orthotopically implanted into the mammary fat pad (*n* = 10/group). Tumor volumes were measured over time; ns, not significant. **b** Tumor weights at day 10 and day 21 post-implantation; bars represent mean ± SEM. **c** Lung metastases were analyzed at day 21 by histological analysis of HE-stained lung sections. Quantitative data are shown as mean ± SEM; statistical tests as indicated. **d** Cytokeratin-positive (CK⁺) tumor cells in inguinal (ILN) lymph nodes were quantified by flow cytometry at indicated time points. Total CK⁺ cell numbers were normalized using counting beads. Statistical analysis was performed using one-way ANOVA. Data are presented as mean ± SEM. **e** Representative immunofluorescence images showing the distribution of blood vessels (CD31, green) and lymphatic vessels (LYVE1, red) in 4T1-vector (left) and 4T1-EGFR (right) primary tumors. Nuclei are stained with Hoechst (blue). The main panel shows the overall vascular and lymphatic structure in the tumor tissue (scale bar = 1000 µm). Regions of interest (ROIs) from the tumor center (yellow box) and tumor periphery (purple box) are enlarged to show the detailed morphology and localization of blood and lymphatic vessels (scale bar = 200 µm).

Orthotopic implantation of 4T1-EGFR or control cells into the mammary fat pad of immunocompetent female BALB/c mice revealed no significant differences in tumor growth kinetics over a 21-day period (Fig. 1a) but 4T1-EGFR tumors displayed slightly lower final weights compared to 4T1-vector controls (Fig. 1b). Histological analysis of lung sections showed no difference in distant metastatic lesions (Fig. 1c and Supplementary Fig. 2a). In contrast, EGFR overexpression significantly increased tumor cell burden in tumor-draining lymph nodes (TDLNs) as revealed by single cell analysis of cytokeratin-positive (CK⁺) cells in inguinal (ILN) and axillary lymph nodes (ALN) at early (day 10) and late (day 21) time points (Fig. 1d and Supplementary Fig. 2b–d).

As a next step, we stained tumor sections from the 4T1 model with markers against blood and lymphatic vessels to visualize where these were located compared to tumor cells. CD31⁺ blood vessels were distributed throughout the tumor parenchyma (Fig. 1e). In contrast, LYVE-1⁺ lymphatic vessels were predominantly located at the tumor periphery, whereas intratumoral lymphatics were rarely detectable. A similar distribution pattern of LYVE-1⁺ lymphatic vessels and CD31⁺ blood vessels was observed in both 4T1-vector and 4T1-EGFR tumor.

Notably, EGFR overexpression did not result in increased expression of lymph node–homing chemokine receptors^19^, which are well-established mediators of lymph node tropism (Supplementary Fig. S3). This indicates that enhanced lymphatic dissemination is not driven by altered chemokine receptor programming at the tumor cell level.

These results indicated that 4T1 cells overexpressing EGFR had an increased capacity to disseminate through peripheral lymphatic vessels.

### Activated lymphatic endothelial cells produce and secrete EGFR ligands

**Fig. 2:**
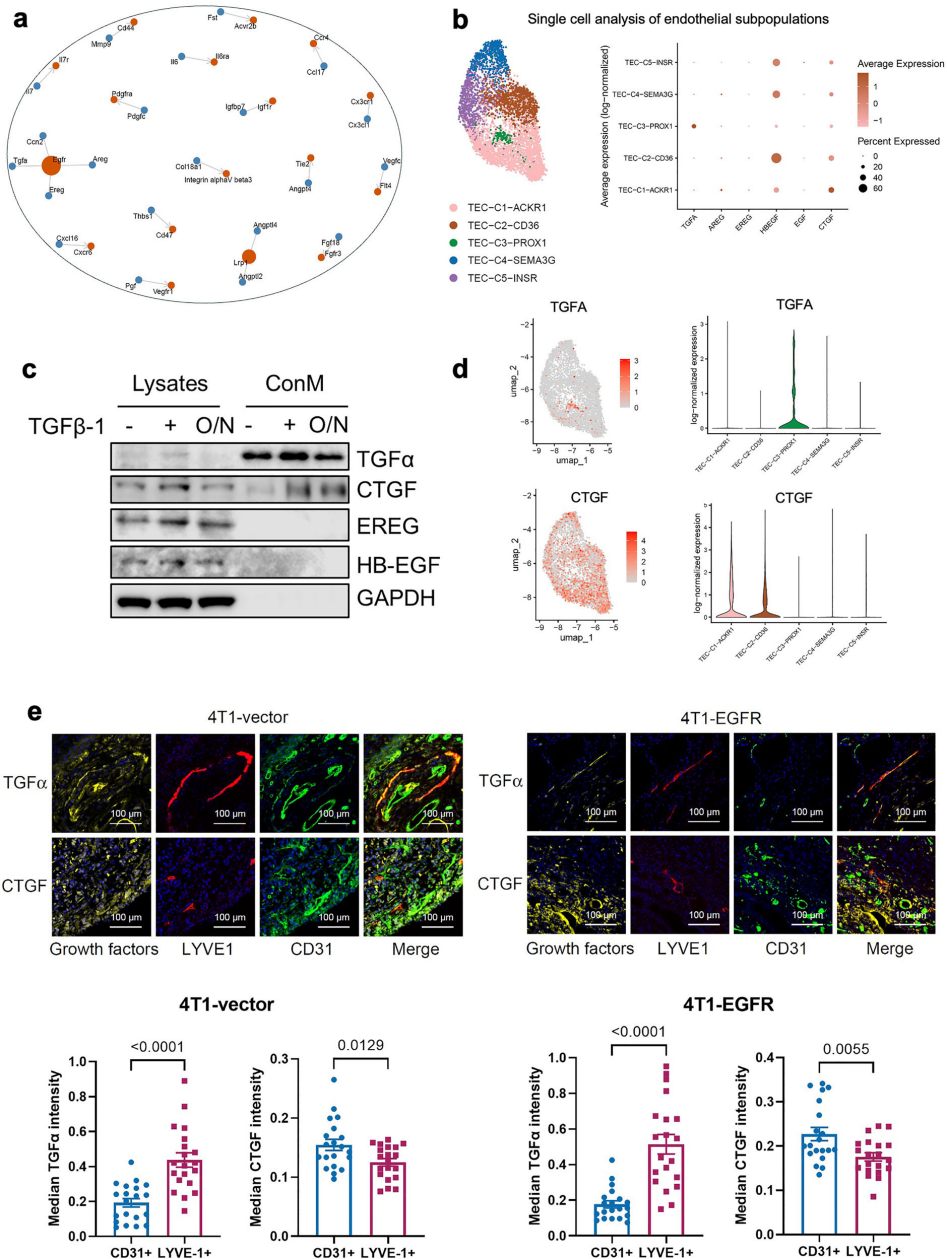
Activated lymphatic endothelial cells produce and secrete EGFR ligands. **a** Genes upregulated upon TGF-β1-mediated activation of svLEC cells and encoding extracellular proteins were analyzed for their involvement in Ligand–receptor interactions using the PhoneDB database. Corresponding receptors are shown in red and ligands in blue. The size of each receptor node reflects the number of interacting ligands. **b** Single-cell RNA-seq analysis using breast cancer and matched metastatic lymph node samples from dataset GSE167036, focusing on the endothelial cell compartment. UMAP visualization revealed five distinct endothelial subpopulations from both primary tumor and LN metastasis, characterized by the expression of ACKR1, CD36, PROX1, SEMA3G, and INSR. Dot plot showing the expression of TGFA, AREG, EREG, HBEGF, EGF, and CTGF across endothelial subpopulations. Dot size represents the percentage of cells expressing each gene, and color intensity indicates average log-normalized expression levels. **c** Western blot analysis of TGFα, CTGF, EREG, and HB-EGF expression in svLEC cell lysates and conditioned media (ConM) following TGF-β1 treatment and after overnight incubation (O/N) in TGF-β1-free medium. GAPDH was used as a loading control for cell lysates. For conditioned media, sample loading was normalized to cell number. **d** Feature plots show the spatial distribution of CTGF and TGFA expression across UMAP. Violin plots illustrate that the expression of TGFα and CTGF is enriched in lymphatic and blood endothelial cells, respectively. Gene expressions are displayed as log-normalized values. **e** Immunofluorescence images showing staining of growth factors (TGFα and CTGF, yellow) in lymphatic (LYVE1+, red) and blood vessels (CD31+, green), respectively, 4T1-vector (left) and 4T1-EGFR (right) tumors (scale bar = 100 µm). Quantification of median TGFα and CTGF signal intensity within regions positive for CD31 and LYVE-1. 20 areas (Regions of Interest ROIs) per group. *n* = 10 per group. Statistical analysis was performed using an unpaired two-tailed Student’s T-test. Data are presented as mean ± SEM.

Based on this, we hypothesized that lymphatic endothelial cells (LECs) might secrete chemotactic cues that specifically attract EGFR-expressing tumor cells, thereby facilitating lymphatic dissemination. To explore this, we re-analyzed RNA-sequencing data from our previous study in which primary murine skin-derived LECs (svLECs) were treated with TGF-β1 to mimic an inflamed or tumor-associated state^17^. Gene ontology and pathway analyses revealed strong induction of genes encoding secreted ligands, several of which are predicted EGFR ligands, including TGF-α, CTGF, EREG, HB-EGF, and AREG (Supplementary Fig. S4a–c). Ligand–receptor network analysis identified EGFR as a central node downstream of the TGF-β1-induced secretome in svLECs (Fig. 2a).

To validate these findings in a clinically relevant setting, we analyzed a single-cell RNA-sequencing dataset from human breast tumors and matched metastatic lymph nodes (GSE167036)^20^. Five transcriptionally distinct populations of ECs were identified, including ACKR1⁺ venous ECs, CD36⁺ capillary subsets, PROX1⁺ lymphatic ECs, SEMA3G^+^ arterial ECs, and INSR^+^ tumor-related ECs enriched in tip cells of angiogenic sprouts (Fig. 2b, d). Dot plot analysis showed that TGFA expression was highest in PROX1⁺ lymphatic endothelial cells, whereas CTGF showed enriched expression in blood-associated ACKR1⁺ and CD36⁺ ECs. The expressions of AREG, EREG, and EGF were negligible across all endothelial clusters, while HB-EGF was expressed in all endothelial cells except PROX1⁺ cells.

To explore whether LECs actually secrete EGFR ligands, we performed western blot analysis of lysates and conditioned medium from untreated and TGF-β1 stimulated SVLEC cells. The results showed that both TGF-α and CTGF were detectable at baseline in lysates and conditioned medium from svLECs, and were further upregulated following TGF-β1 stimulation (Fig. 2c). EREG and HB-EGF were similarly induced in the lysates but were not detected in the conditioned medium. TGF-α remained present in conditioned medium even after TGF-β1 withdrawal, supporting its potential to act as a paracrine chemoattractant.

Together, these findings indicated that TGF-α and possibly CTGF could be candidate EGFR ligands, which may guide EGFR-overexpressing tumor cells into lymphatic vessels.

### TGF-α localizes to the lymphatic endothelium in vivo and predicts poor prognosis

To understand the spatial distribution of EGFR ligands in vivo, we examined the localization of TGF-α and CTGF within the tumor vasculature of orthotopic 4T1 tumors. TGF-α was enriched in LYVE1⁺ lymphatic vessels, whereas CTGF predominantly localized to CD31⁺ blood vessels (Fig. 2e). This differential localization was consistent across 4T1-vector and 4T1-EGFR tumor sections, suggesting that TGF-α may serve as a spatially restricted chemotactic cue for lymphatic entry.

In support of a clinical relevance for TGFA in basal-like breast cancer, we found that the expression levels of TGFA were the highest in these tumors compared to luminal A or luminal B tumors, as defined by the PAM50 gene signature classification (Supplementary Fig. S5a, b). Furthermore, we found that high expression of TGFA—but not CTGF—expression was associated with poor survival in human breast cancer, specifically in basal-like tumors (Supplementary Fig. S6). Taken together, these data support a model in which TGF-α–producing lymphatic endothelial cells create a directional chemotactic gradient sensed by EGFR-overexpressing tumor cells, facilitating selective lymphatic dissemination.

### EGFR mediates chemotactic migration toward lymphatic-derived TGF-α

**Fig. 3:**
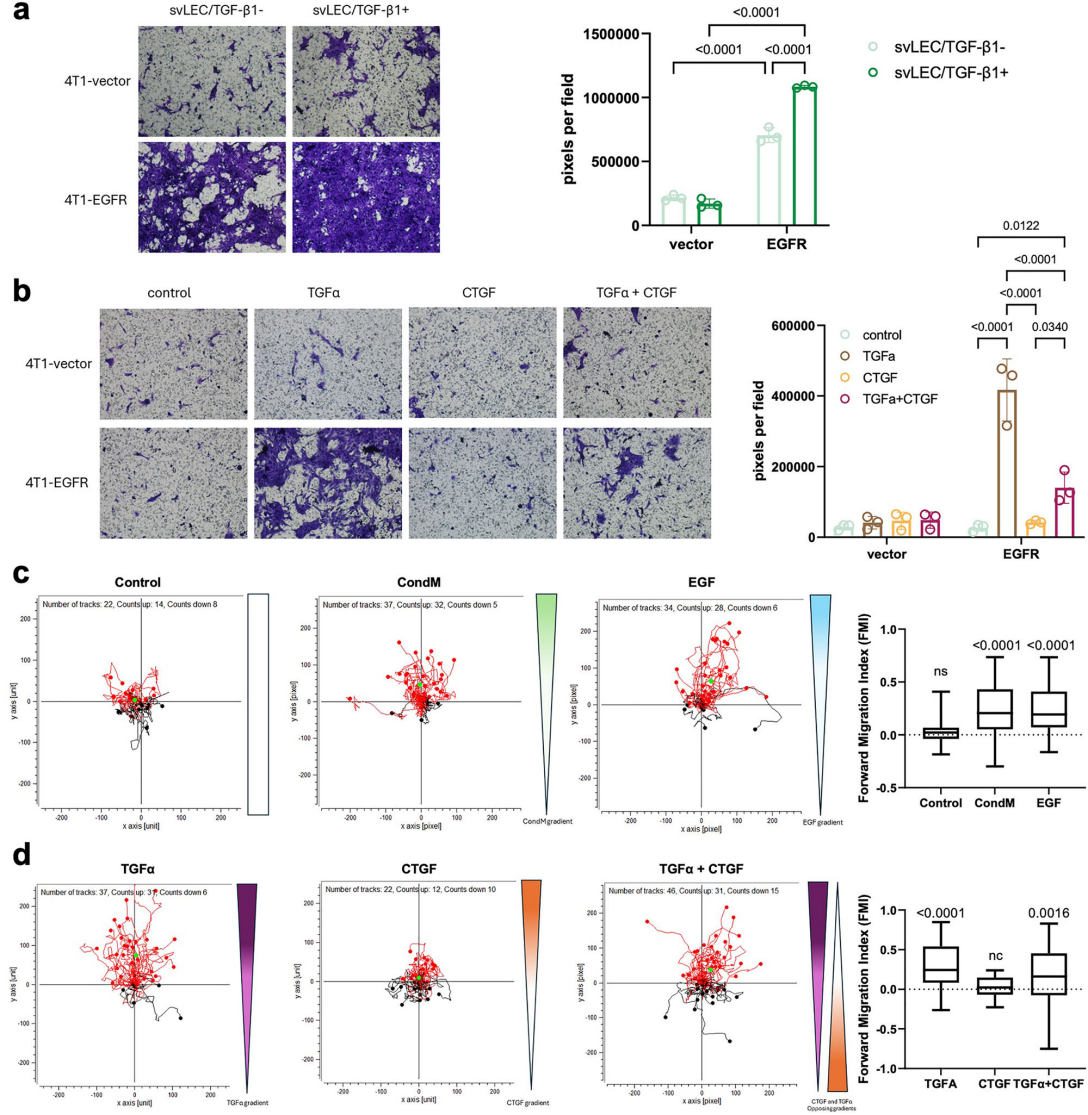
EGFR mediates directional migration of tumor cells toward lymphatic signals. Transwell invasion assays of 4T1-vector and 4T1-EGFR cells toward conditioned medium (CondM) from svLECs, with or without prior TGF-β1 stimulation (**a**), or serum-free medium supplemented with recombinant TGF-α, CTGF, or both (**b**). Three fields per well were analyzed across three independent experiments by quantifying the crystal violet-stained area. Statistical analysis was performed using two-way ANOVA. **c**, **d** Directional migration of 4T1-EGFR cells was assessed using chemotaxis assays and quantified by the Forward Migration Index (FMI). The significance of vectorial migration was evaluated using the Rayleigh test. As indicated by the dashed line, each condition is assessed relative to its own “zero directionality” reference. **c** Chemotactic responses to svLEC-conditioned medium (CondM) were examined, with control medium and EGF serving as negative and positive controls for assay performance. **d** Directed migration in response to defined EGFR ligands was assessed using gradients of CTGF or TGFα. In the combined condition, CTGF and TGFα were placed in opposing reservoirs to model competitive chemotaxis.

To assess whether EGFR can function as a chemotactic receptor guiding tumor cells toward lymphatic-derived signals, we used a series of migration assays. As a first step, we tested whether conditioned medium from lymphatic endothelial cells (LECs) could promote directed movement of EGFR-expressing tumor cells. In transwell invasion assays, 4T1-EGFR cells displayed markedly increased invasion toward conditioned medium (CondM) from unstimulated svLECs, and this response was further enhanced when LECs were pre-treated with TGF-β1 (Fig. 3a). In contrast, vector control cells showed minimal invasion under all conditions. This pattern was mirrored in wound healing assays, in which exposure to TGF-β1–activated LECs-conditioned medium significantly accelerated wound closure in 4T1-EGFR cells compared to control LECs-conditioned medium. While 4T1-vector cells showed no significant response to TGF-β1–activated LEC-conditioned media (Supplementary Fig. 7a). These findings suggested that the lymphatic secretome provides directional cues that are selectively recognized by EGFR-overexpressing tumor cells.

To dissect the ligand contribution, we supplemented serum-free medium with recombinant TGF-α, CTGF, or both. TGF-α alone elicited a strong invasive and migratory response in 4T1-EGFR cells, while CTGF alone had a suppressive or neutral effect (Fig. 3b and Supplementary Fig. 7b). Co-treatment with TGF-α and CTGF produced an intermediate—but still significant—response compared to TGF-α alone, suggesting that CTGF may partially antagonize TGF-α–driven motility.

We next employed a live-cell chemotaxis assay to assess directional migration. 4T1-EGFR cells showed potent vectorial migration toward LEC-CondM, EGF, and TGF-α, respectively (Fig. 3c, d). CTGF, by contrast, did not induce directional motility. When CTGF and TGF-α were applied to opposite reservoirs, cells continued to migrate toward the TGF-α source with unchanged speed and persistence, demonstrating that TGF-α dominates the chemotactic response even in the presence of CTGF. These results establish that EGFR functions as a chemotactic receptor in breast cancer cells, enabling directional migration toward lymphatic-derived TGF-α. While CTGF may modulate the motility state, our data indicate that TGF-α provides the dominant guidance signal driving lymphatic invasion.

### EGFR overexpression activates STAT3 signaling downstream of lymphatic signals

**Fig. 4:**
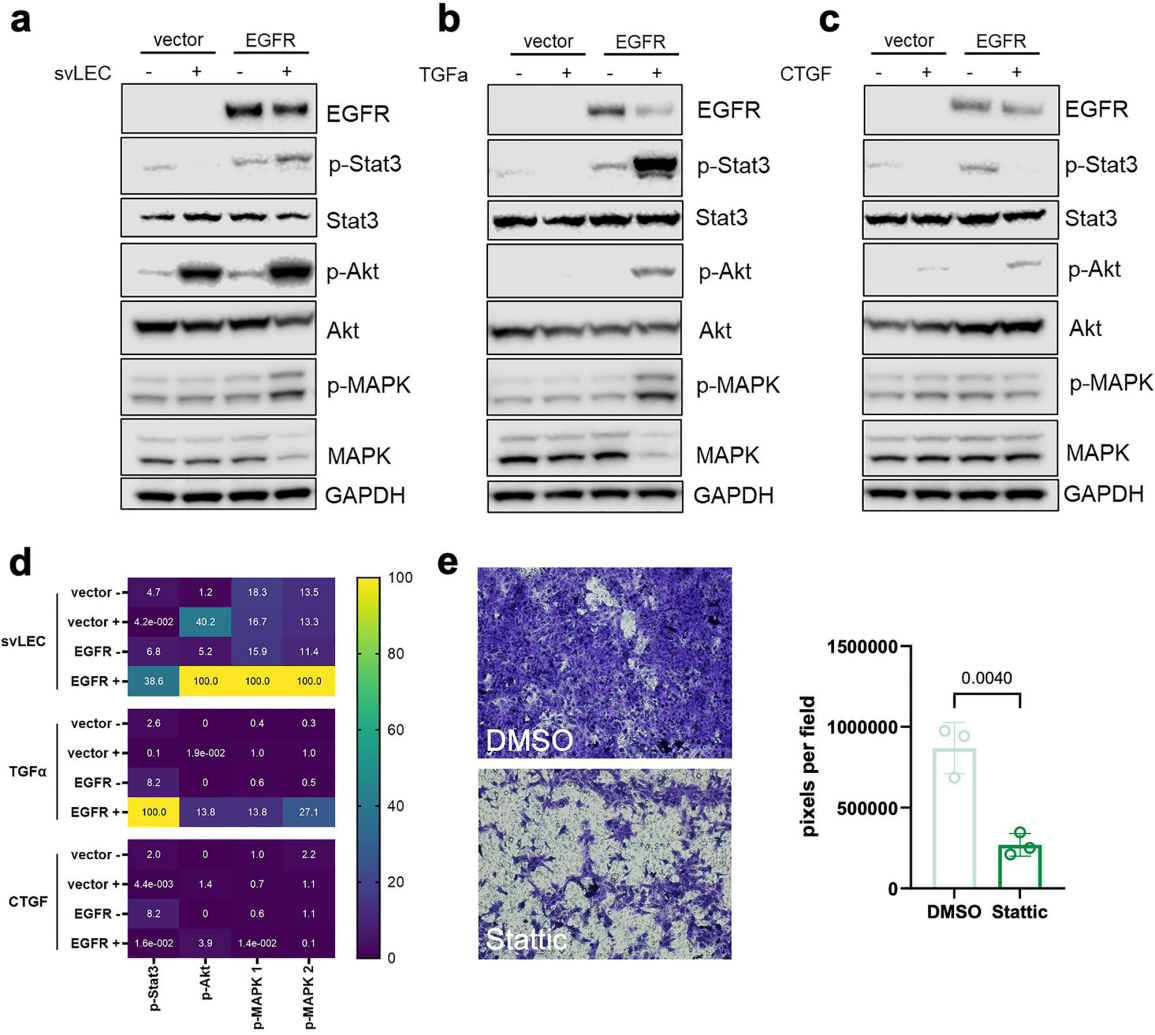
Chemotactic invasion of EGFR-expressing tumor cells toward lymphatic signals is mediated through STAT3-dependent signaling. **a**–**c** Western blot analysis of the effect of svLEC-conditioned medium (**a**, svLEC), TGF-α (**b**), or CTGF (**c**) on phosphorylated and total proteins in the EGFR signaling pathway. Expression levels of phosphorylated and total STAT3, MAPK, and AKT were assessed. GAPDH was used as a loading control. **d** Signal intensities were quantified using ImageJ, and phosphorylation levels were normalized to the corresponding total protein and further to GAPDH. All targets were probed on the same membrane per experiment. Results from three independent biological replicates were quantified. Normalized values were processed in GraphPad Prism and visualized as a heatmap. **e** The STAT3 inhibitor Stattic was used to assess the role of STAT3 signaling in tumor cell invasion. Transwell invasion assay showing the invasive capacity of 4T1-EGFR cells toward svLEC-conditioned media in the presence of Stattic. DMSO was used as vehicle control. For each condition, three fields per well were imaged and quantified across three independent experiments based on crystal violet-stained areas. Statistical analysis was performed using an unpaired two-tailed Student’s T-test. Data are presented as mean ± SEM.

To dissect the signaling mechanisms underlying EGFR-driven lymphatic metastasis, we compared the intracellular responses of 4T1-EGFR cells to different extracellular stimuli. Immunoblotting revealed that EGF stimulation of 4T1-EGFR cells induced phosphorylation of STAT3 (Supplementary Fig. 8), which was not evident in 4T1-vector cells, showing the functional capacity of the overexpressed EGFR to activate intracellular signaling upon ligand binding. We then assessed the effect of conditioned medium from svLECs and found that it also induced phosphorylation of STAT3 in 4T1-EGFR cells compared to 4T1-vector cells, and also activated ERK1/2 (MAPK), and AKT, indicating activation of multiple downstream pathways (Fig. 4a). Stimulation with recombinant TGF-α resulted in a similar type of broad activation pattern, but with a stronger effect on pSTAT3 relative to pERK (Fig. 4b). In contrast, CTGF induced phosphorylation of AKT alone, without activating STAT3 or MAPK (Fig. 4c). Quantitative analysis from three biological replicates confirmed that only svLEC conditioned medium and TGF-α significantly increased pSTAT3 and pERK levels **(P** < **0.01)**, while all conditions elevated pAKT to a similar degree (Fig. 4d).

These results suggest that TGF-α induces directional migration via coordinated activation of STAT3 and MAPK, whereas CTGF, despite activating AKT, provides a restricted, non-chemotactic signal. Together, the LEC-derived secretome integrates TGF-α–mediated motility with CTGF-induced survival or adhesion signals, forming a specialized lymphatic niche that supports EGFR⁺ tumor cell trafficking. To directly assess the role of STAT3, we treated cells with Stattic, a selective small-molecule STAT3 inhibitor^21^. In Transwell assays, Stattic treatment nearly abolished 4T1-EGFR cell invasion toward LEC-conditioned medium (Fig. 4e), confirming that STAT3 activation is essential for EGFR-mediated lymphatic chemotaxis.

### EGFR-dependent directional migration toward TGF-α is conserved in human basal-like breast cancer cells

**Fig. 5:**
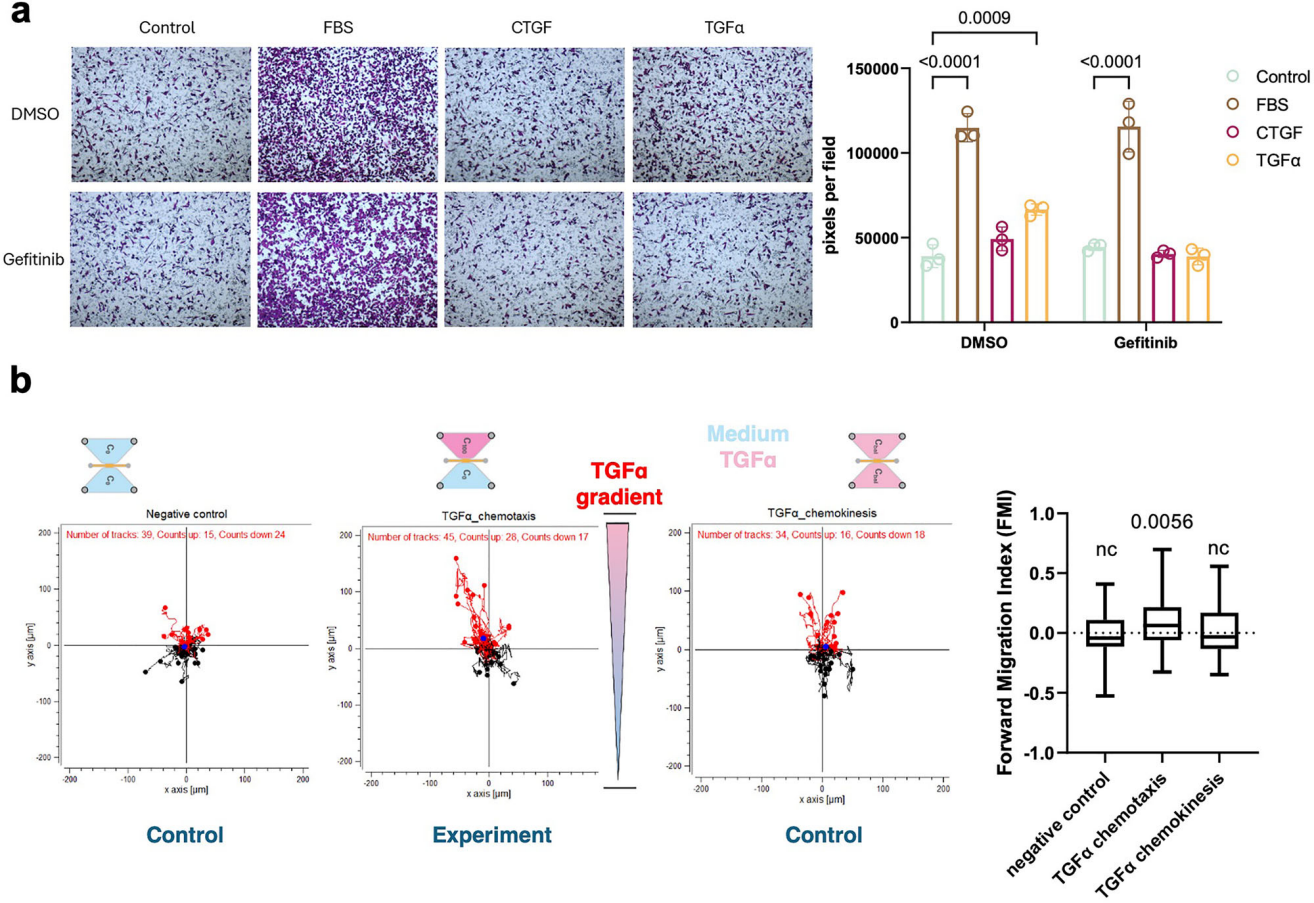
EGFR-dependent directional migration of MDA-MB-231 cells toward TGFα. **a** Results from invasion assays demonstrating the invasive capacity of MDA-MB-231 cells toward serum-free media supplemented with FBS, TGFα, or CTGF. Gefitinib was added to assess the role of EGFR signaling. DMSO was used as a control. Crystal violet staining pixel areas were quantified, and statistical analysis was performed using two-way ANOVA. **b** Chemotaxis assay showing the directed migration of MDA-MB-231 cells toward TGFα. Forward Migration Index (FMI) was quantified and displayed as a bar plot. Directionality was assessed using the Rayleigh test based on migration endpoints.

To test whether our findings in the murine 4T1 model extend to human breast cancer, we employed the triple-negative MDA-MB-231 cell line, which represents a basal-like subtype and expresses high levels of endogenous EGFR, as shown (Supplementary Fig. S1a). Using transwell invasion assays, we assessed the capacity of MDA-MB-231 cells to invade toward serum-free medium supplemented with individual EGFR ligands. As in the murine system, TGF-α robustly induced invasive migration of MDA-MB-231 cells, whereas CTGF had no stimulatory effect and trended toward suppression (Fig. 5a). The positive control (FBS) confirmed that cells retained general chemotactic responsiveness. To investigate the requirement for EGFR signaling, we treated cells with gefitinib, a clinically approved EGFR tyrosine kinase inhibitor. Gefitinib significantly suppressed the invasion of MDA-MB-231 cells toward TGF-α, restoring invasive capacity to baseline levels (*P* < 0.0001 vs. DMSO-treated cells). This result indicates that the observed migration is dependent on active EGFR signaling.

To further confirm that TGF-α provides a directional chemotactic cue rather than a general motogenic stimulus, we performed live-cell chemotaxis assays. MDA-MB-231 cells exhibited a strongly positive Forward Migration Index (FMI) toward TGF-α, indicating directional migration (Fig. 5b).

These data demonstrate that EGFR functions as a bona fide chemotactic receptor in human basal-like breast cancer cells, with TGF-α acting as a potent guidance cue. Importantly, this function is susceptible to pharmacological inhibition, supporting the concept of repositioning EGFR inhibition to target lymphatic dissemination rather than primary tumor growth in breast cancer.

### Therapeutic TGF-α blockade impairs lymphatic metastasis

Having identified TGF-α as the principal chemotactic driver of EGFR-directed lymphatic metastasis, we next asked whether blocking this ligand would interrupt lymph metastasis. We used Fepixnebart (LY3016859), a humanized IgG4 monoclonal antibody with high affinity and neutralizing activity for TGF-α and epiregulin^22,23^. Fepixnebart has undergone early-phase clinical evaluation in patients with chronic pain^24^ and has shown favorable safety and on-target activity^25^. However, it has so far not been tested in tumor studies.

**Fig. 6:**
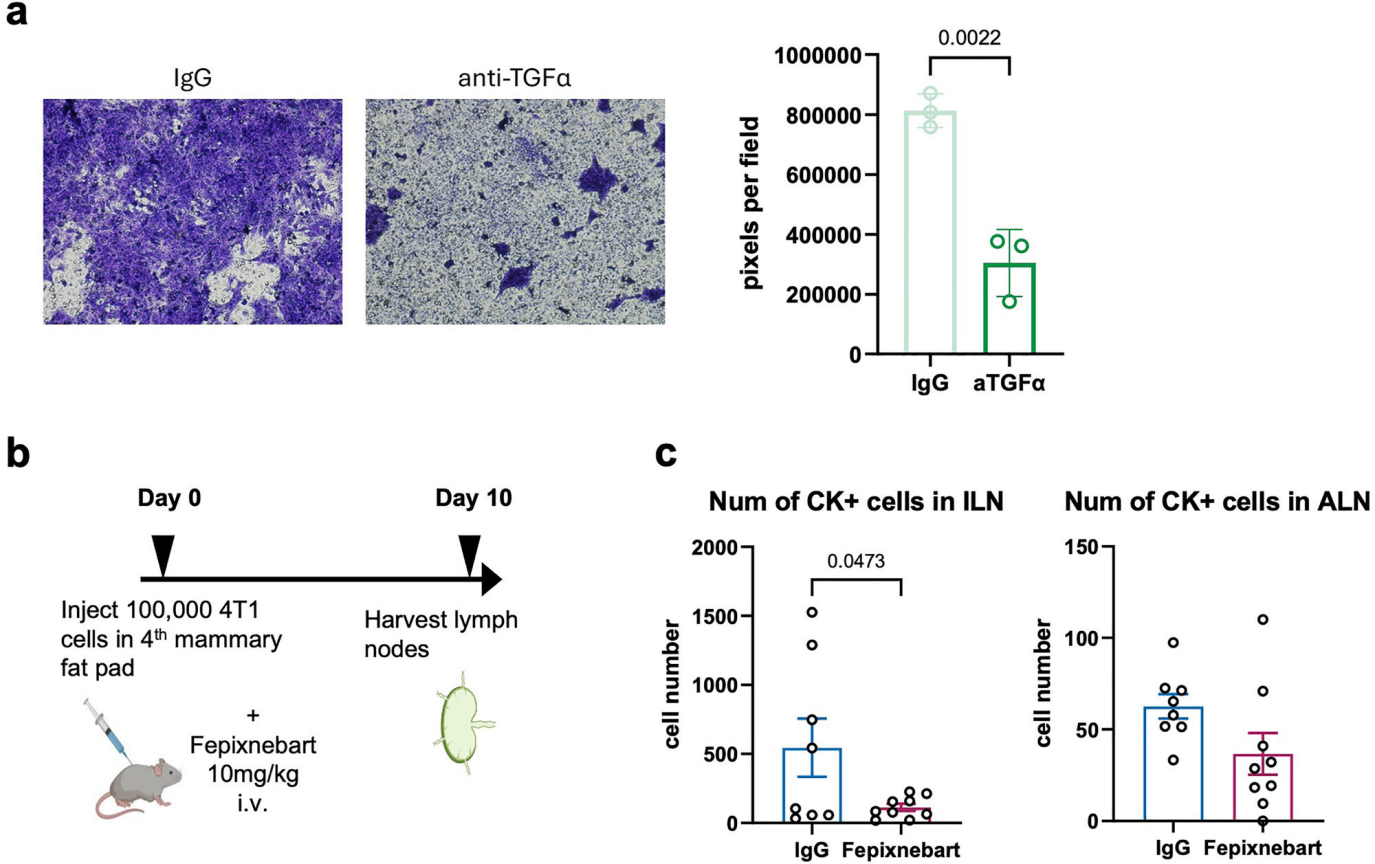
Fepixnebart suppresses TGFα-mediated invasion and lymph node metastasis of 4T1-EGFR cells. Studies were performed to analyze whether blockade of TGF-α could inhibit chemotactic invasion and lymphatic metastasis of 4T1-EGFR cells. **a** Results from transwell invasion assays showing that Fepixnebart, a neutralizing antibody against TGFα (aTGFα), significantly inhibited chemotactic invasion of 4T1-EGFR cells toward svLEC-conditioned media. An isotype matched IgG antibody was used as control. For each condition, three fields per well were imaged and quantified across three independent experiments based on crystal violet-stained areas. Statistical analysis was performed using an unpaired two-tailed Student’s T-test. **b** Schematic overview of the in vivo experimental setup. 4T1 cells were orthotopically implanted into mice, followed by tail vein injection of Fepixnebart. Tumor-draining lymph nodes were collected for analysis 10 days later. **c** Bar graphs showing quantification of CK⁺ tumor cells in inguinal (ILN) and axillary (ALN) lymph nodes 10 days after orthotopic injection of 4T1-EGFR cells into mammary fat pads. Mice received a single intravenous injection of Fepixnebart (10 mg/kg; *n* = 9) or isotype IgG control (*n* = 8) at the time of tumor cell implantation. Data are shown as mean ± SEM; statistical analysis by unpaired two-tailed Student’s t-test.

To determine whether Fepixnebart directly disrupted TGF-α-driven chemotactic migration of EGFR expressing tumor cells, we repeated transwell invasion assays. Fepixnebart markedly reduced the invasion of 4T1-EGFR cells toward LEC-conditioned medium compared to the effect induced by control IgG antibody (Fig. 6a). Based on published pharmacokinetic profiles, we administered a single intravenous dose of Fepixnebart (10 mg/kg) at the time of orthotopic implantation of 4T1-EGFR cells. Ten days later, mice treated with Fepixnebart displayed a significant reduction in cytokeratin-positive (CK⁺) tumor cell burden in the ILN compared to control mice receiving isotype IgG (Fig. 6b, c). Tumor cell counts in ALN showed a similar trend but did not reach significance.

These results provide preclinical proof-of-concept that TGF-α blockade can intercept early lymphatic metastasis driven by EGFR—offering a therapeutic repositioning strategy for EGFR antagonists in breast cancer.

### EGFR is associated with enhanced cellularity and an immunoregulatory compositional shift in tumor-draining lymph nodes

**Fig. 7:**
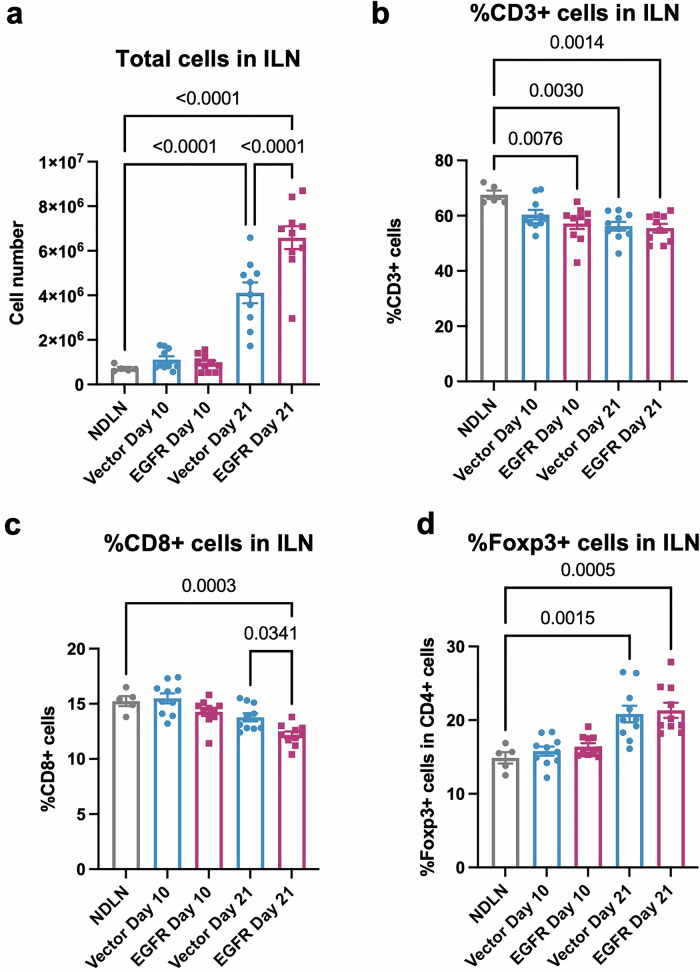
Cellularity and an immunoregulatory compositional shift in tumor-draining lymph nodes. **a** Total number of cells in inguinal lymph nodes (ILN) compared to non-draining lymph node (NDLN) at day 10, and day 21 following orthotopic implantation of 4T1-vector or 4T1-EGFR cells. Percentage of CD3⁺ T cells (**b**), CD8 + T cells (**c**), and Foxp3⁺ regulatory T cells (**d**) in ILN compared to NDLN at day 10, and day 21 following orthotopic implantation of 4T1-vector or 4T1-EGFR cells. Each dot represents one lymph node; group means ± SEM shown. Statistical analysis was performed by two-way ANOVA with post hoc test.

Finally, to examine whether EGFR-associated lymphatic involvement is accompanied by changes in immune architecture in regional lymph nodes, we profiled immune cell compositions in TDLNs at an early (day 10) and a later (day 21) time point following orthotopic injection of 4T1-vector or 4T1-EGFR cells. In both ILN and ALN, total cell numbers were significantly increased at day 21 compared to non-draining lymph nodes (NDLNs) (Fig. 7a**;** Supplementary Fig. S10a). Notably, TDLN cellularity was consistently higher in the EGFR-overexpressing group than in vector controls at both time points, indicating a more pronounced lymph node response in the presence of EGFR overexpression.

Despite the increased cellularity, the frequency of CD3⁺ T cells in ILNs was reduced in the 4T1-EGFR group on day 10 when compared with NDLNs; this decrease was most evident in ILNs and was not observed in ALNs (Fig. 7b; Supplementary Fig. S10b). On day 21, the frequency of CD3⁺ T cells was decreased in both groups compared to NDLNs. Within the T-cell compartment, CD4⁺ T-cell frequencies remained largely stable across the groups and time points (Supplementary Fig. S10c, d). In contrast, CD8⁺ T cells showed a selective reduction in ILNs rather than ALNs in the EGFR group at day 21 relative to NDLNs, and CD8⁺ T-cell representation in ILNs was also lower in the EGFR group than in vector controls (Fig. 7c; Supplementary Fig. S10e). Regulatory T cells (Tregs; Foxp3⁺) increased over time in both ILNs and ALNs of tumor-bearing mice, with significantly higher frequencies in both groups at day 21 compared with NDLNs (Fig. 7d; Supplementary Fig. S10f).

Together, these data indicate that tumor drainage is associated with progressive immune compositional remodeling in TDLNs, characterized by increased cellularity and a T-cell compartment skewed toward lower CD8⁺ representation with increased Tregs over time. Within the immune subsets assessed here, EGFR-associated differences were most evident in enhanced TDLN cellularity and reduced CD8⁺ T-cell representation at the later time point, consistent with an immunoregulatory compositional shift in TDLNs accompanying EGFR overexpression, while not by themselves defining functional immune exhaustion or suppression.

## Discussion

Our findings that EGFR overexpression drives lymph node metastasis in a murine triple-negative breast cancer model add novel insight to the role of EGFR in cancer progression. While EGFR has long been recognized for its contribution to tumor aggressiveness through enhanced proliferation, survival, and invasiveness, its specific role in facilitating lymphatic dissemination has remained largely underexplored.

Clinical pathology studies have reported that EGFR overexpression in primary breast tumors correlates with increased lymph node involvement, especially in basal-like and triple-negative breast cancer subtypes^26^. However, the mechanistic basis for this association has remained unclear. Previous studies have primarily focused on the role of EGFR in hematogenous dissemination and organ colonization, often in models of lung metastasis. The possibility that EGFR could play a distinct, non-proliferative role in guiding tumor cells into the lymphatic system has not been systematically tested.

The paradoxical functions of EGFR in breast cancer have been discussed, and it has been suggested that the outcome of EGFR signaling is context-dependent on downstream effectors and ligand availability^27^. Yet, lymphatic metastasis was not highlighted in that discussion. In contrast, our study provides direct experimental evidence that EGFR promotes lymph node metastasis without affecting tumor growth rate or lung metastatic burden. This selective enhancement of lymphatic spread indicates a migration-specific function of EGFR in certain microenvironmental contexts, independent of its proliferative signaling.

We identified LECs as a previously unrecognized source of TGF-α within the breast tumor microenvironment and demonstrated that TGF-α acts as a potent directional cue for EGFR-expressing tumor cells. Lymphatic vessels were located at the peripheral parts - rather than in the parenchyma of 4T1 tumors, consistent with previous results showing that high interstitial pressures in rapidly growing tumors result in collapsed lymph vessels inside of the tumors^28^. These observations support that primary 4T1 tumor cells metastasize to lymph nodes through peripheral lymph vessels and that tumor cells may require additional directional migration capacity to reach these peripheral lymphatics. This notion is consistent with our proposed model, in which growth factors secreted by peritumoral lymphatics, such as TGF-α, provide chemotactic cues that facilitate the guidance of tumor cells toward accessible lymphatic vessels and thereby promote lymphatic dissemination.

These findings add a new dimension to the role of lymphatic vessels in metastasis, extending beyond passive conduits or mechanical barriers to active participants in guiding tumor cell migration. Previous studies have shown that breast cancer cells can reprogram LECs to support tumor growth and metastasis through pro-angiogenic and immunomodulatory signaling cascades^29,30^. LECs have been implicated in creating pre-metastatic niches and supporting tumor cell adhesion, transmigration, and immune suppression^29–31^. However, the molecular mediators of direct LEC tumor cell chemotactic communication have remained elusive.

Lymph node metastasis is a multistep process that encompasses local invasion, lymphatic entry, intralymphatic trafficking, and subsequent colonization of the lymph node^32^. Our results show that LECs basally express and secrete TGF-α and that this expression is further induced by TGF-β1, a cytokine frequently enriched in aggressive breast tumors. The resulting secreted TGF-α acts as a potent chemotactic ligand for EGFR-overexpressing tumor cells. Importantly, the enhanced lymphatic dissemination observed in EGFR-overexpressing tumor cells cannot be attributed to increased expression of classical lymph node–homing chemokine receptors. Instead, our data support a model in which EGFR signaling acts at an earlier step of the metastatic cascade by facilitating lymphatic engagement, rather than by reprogramming tumor cell homing within the lymph node microenvironment.

In vitro assays revealed that TGF-α alone is sufficient to drive directional migration, while CTGF, another EGFR ligand upregulated in the vasculature, does not support directional motility and may instead serve to modulate or restrain TGF-α-induced responses. While extended time-course analyses may further clarify whether CTGF-mediated modulation influences cumulative migratory outcomes, our data indicate that, within the experimental timeframe examined, directional guidance is dictated by TGF-α. TGF-α has previously been shown to promote migration of epithelial cells via EGFR activation in corneal epithelial cells^33^, but to our knowledge, this is the first demonstration that lymphatic-derived TGF-α can serve as a spatially localized, paracrine chemoattractant in the context of cancer metastasis. Our data also highlight that this TGF-α-driven chemotaxis is functionally dominant over other stimuli present in the microenvironment, establishing a clear directional axis toward the lymphatic vasculature. Taken together, these findings support a new model in which tumor-secreted TGF-β acts on LECs to stimulate the production of EGFR ligands, most importantly TGF-α, which in turn creates a chemotactic gradient sensed by EGFR-expressing tumor cells. This paracrine signaling axis amplifies lymphatic dissemination and highlights a spatially organized tumor-lymphatic interaction that could be therapeutically targeted.

Our data further revealed that among the pathways activated downstream of LEC-derived signals, STAT3 phosphorylation is most closely associated with directional chemotactic responses and lymphatic invasion. Conditioned medium from lymphatic endothelial cells, as well as recombinant TGF-α, robustly activated STAT3 and ERK1/2 in EGFR-overexpressing tumor cells. However, blockade of STAT3 signaling using the small-molecule inhibitor Stattic was sufficient to abrogate migration toward the lymphatic secretome, suggesting that STAT3 is not merely co-activated but is functionally essential for EGFR-guided chemotaxis. This finding aligns with prior reports implicating STAT3 in metastatic behavior across multiple cancer types. STAT3 activation has been associated with increased cell motility and invasion through the induction of EMT-related genes, cytoskeletal remodeling, and matrix-degrading enzymes^34^. In breast cancer specifically, constitutive STAT3 activity promotes tumor progression and is frequently observed in triple-negative tumors, where it correlates with poor prognosis and therapy resistance^35^. Furthermore, STAT3 signaling has been shown to regulate immune escape by promoting Treg expansion, M2 macrophage polarization, and suppression of effector T cell activity, all of which are central to premetastatic niche formation and lymphatic spread^36^.

In comparison, our data showed that CTGF, despite triggering AKT phosphorylation, fails to drive directional migration, suggesting that AKT activation alone is insufficient to support lymphatic tropism. Similarly, MAPK activation by itself is not dominant in the absence of STAT3 signaling. These observations underscore a degree of pathway specificity in how EGFR signaling translates into chemotactic versus proliferative outputs, and position STAT3 as a pivotal effector of EGFR-mediated lymphatic metastasis in breast cancer.

By targeting TGF-α with the neutralizing antibody Fepixnebart, we were able to significantly reduce tumor cell invasion toward LEC-derived secretome in vitro and inhibit early lymph node metastasis in vivo. These findings suggest that ligand blockade may provide a more effective and context-specific approach for halting lymphatic dissemination in EGFR-positive breast cancer. They further open up for repositioning EGFR-targeted therapies not to suppress proliferation but to interfere with early metastatic events - specifically, directional chemotaxis toward the lymphatic system. This is the first study, to our knowledge, to demonstrate that TGF-α can be effectively targeted to suppress lymphatic metastasis, and the first to use Fepixnebart for this purpose in a preclinical tumor model. Fepixnebart is a first-in-class monoclonal antibody that neutralizes TGF-α and epiregulin with high specificity. It has been evaluated in phase I clinical trials for chronic pain and inflammatory conditions^25^, where it showed favorable safety and on-target effects, but it has not yet been tested in oncology trials. Given its tolerability and specificity, Fepixnebart may offer a clinically actionable route to selectively target the tumor - LEC axis in EGFR-positive breast cancer.

Our immune profiling in TDLNs captured compositional immune remodeling rather than a comprehensive definition of functional immune suppression or T-cell exhaustion. Immunosuppression is a multifaceted phenomenon that depends not only on immune-cell abundance, but also on cellular states and functions (e.g., activation/exhaustion programs, proliferative capacity, cytokine production, and antigen-specific responses). Within this framework, the reduced representation of CD3⁺/CD8⁺ T cells together with increased Foxp3⁺ regulatory T cells is most appropriately interpreted as an immunoregulatory shift with suppressive features in TDLNs^37^. which is consistent with a suppressive trend reported in previous studies of pre-metastatic lymph nodes^38,39^.

A second consideration is that the TDLN-NDLN contrast is strong in both vector and EGFR conditions, consistent with a dominant tumor-drainage effect on lymph node immune composition that can overshadow more subtle genotype-dependent differences when comparing EGFR to vector at the same time point. Against this background, the most consistent EGFR-associated signals in our dataset are increased TDLN cellularity and a selective reduction in CD8⁺ T-cell representation, suggesting that EGFR may modulate the magnitude and/or kinetics of lymph node remodeling rather than broadly reshaping all major immune compartments at the resolution assessed here.

Although EGFR overexpression is associated with earlier alterations in draining lymph nodes in this model, the current data do not by themselves resolve causality or directionality; rather, they support a temporal association between enhanced lymphatic involvement and immune compositional changes. Mechanistically, at least two non–mutually exclusive scenarios could explain these observations. First, lymph node remodeling may precede overt metastatic establishment through chemotaxis-independent pathways, for example, via tumor-derived soluble factors and/or extracellular vesicles reaching tumor-draining/sentinel nodes through lymphatic flow and conditioning the local immune landscape^38,40,41^. Second, lymph node changes may also arise as a consequence of lymph node seeding/colonization itself, which can induce immune tolerance and promote metastatic progression, suggesting potentially bidirectional coupling between lymph node involvement and immune remodeling^11^. Accordingly, the lymph node findings are most appropriately interpreted as EGFR overexpression being associated with, and accompanied by, early immune compositional remodeling in tumor-draining lymph nodes, without implying a resolved causal sequence.

Taken together, the data suggest that the EGFR–TGF-α–LEC axis could be a novel therapeutic target in breast cancer. Ligand-directed intervention using Fepixnebart offers a new rationale for EGFR targeting, one that is spatially and mechanistically linked to lymphatic dissemination and immune suppression. Future clinical trials should explore TGF-α blockade both as monotherapy to prevent lymphatic metastasis and as a combinatorial approach to improve the efficacy of immune-based treatments.

## Methods

### Cell culture

4T1 cells were from ATCC (American Type Culture Collection, VA) and cultured in RPMI and supplemented with 10% Fetal Bovine Serum (FBS, Thermo Fisher Scientific) and 1% penicillin + streptomycin. Mouse lymphatic endothelial cells (svLEC) (provided by Dr Jonathan S. Alexander, Louisiana State University Health Sciences Center, Shreveport, Louisiana), and human MDA-MB-231 and MCF-7 breast cancer cells (both from ATCC) were cultured in DMEM with high glucose supplemented with 10% FBS and 1% penicillin–streptomycin. All cells were cultured in 37 °C and 5% humidified CO_2_ incubator.

### Preparation of svLEC-conditioned medium

svLECs were seeded in T-75 flasks at 2 × 10⁶ cells in complete RPMI-1640 medium and allowed to adhere overnight. The next day, the cultures were washed twice with PBS and replaced with 10 mL serum-free RPMI-1640. After 48 h, supernatants were harvested, centrifuged (500 × *g*, 5 min, 4 °C) to remove cell debris, aliquoted, and stored at −80 °C for downstream assays. For cytokine-profiling experiments involving TGF-β stimulation, cells were first exposed to complete medium containing 10 ng/mL recombinant TGF-β1 (7754-BH-025/CF, R&D Systems) for 48 h immediately after overnight attachment. The stimulus was then removed, fresh serum-free medium was added, and conditioned medium was collected after a further overnight incubation following the same procedure described above.

### Generation of 4T1-EGFR cells using the piggyBac system

4T1 cells were co-transfected with a PiggyBac Transposase plasmid and a plasmid containing either the stuffer sequence (4T1-vector) or Egfr (NM_207655.2) (4T1-EGFR), both with a puromycin resistance marker. Transfection was performed using the SE Cell Line 4D-Nucleofector X Kit S (V4XC-1032, Lonza) and the Nucleofector 4D, X-unit (AAF-1002X, Lonza), according to the manufacturer’s instructions. The CM-150 electroporation program was applied to 4T1 cells. After transfection, cells were selected by puromycin for 2 weeks. The resulting pools of 4T1 cells were then validated for gene overexpression by RT-qPCR and Western blotting.

### Animal care

Wild-type 8-week-old female mice with a Balb/c background were used. The mice were housed in a standard animal facility (KMB, Karolinska Institutet), housed in groups of up to 5 animals per cage in IVC-500 cages, under controlled temperature (22 °C) and photoperiod (12 h of light, 12 h of dark), and given water and pellet diet (CRM(P), Special Diet Services, Scanbur). All animal experiments were approved by Karolinska Institutet and the Stockholm Regional Board for Animal Ethics (Ethical numbers: 11055-2023 with amendment 2-2128/2024) and complied with the ARRIVE guidelines. Mice were anesthetized with isoflurane delivered via inhalation using a vaporizer system. Analgesia was provided with buprenorphine (0.1 mg/kg, subcutaneous) when required according to the approved animal ethics protocol. Animals were euthanized under deep isoflurane anesthesia followed by cervical dislocation. Cohorts were chosen to ensure reproducibility and to allow stringent statistical analysis.

### Generation of the 4T1-EGFR tumor model to assess lymphatic metastasis

Female mice were randomly assigned to experimental groups at the time of tumor cell implantation and injected with 1 × 10^5^ 4T1-vector or 4T1-EGFR cells in 5 μl PBS into the fourth mammary fat pad. Mice were euthanized, and tumor-draining lymph nodes were harvested on day 10 and day 21. Single-cell suspensions of tumor-draining lymph nodes were obtained after digestion in RPMI supplemented with 150 µg/ml Liberase (Roche), 50 µg/ml DNase I (Roche), and incubated with the FcR Blocking Reagent (Miltenyi Biotec) to block unspecific binding. For tumor quantification, surface antigens were stained with the following specific antibodies: PerCP-Vio700-anti-CD45 (130-110-801, clone REA737; Miltenyi Biotec), BV605-anti-CD11b (101237, clone M1/70, Biolegend). Cells were fixed, permeabilized, and stained by FITC-anti-pan-cytokeratin (MA5-28561, clone C-11, Thermo Scientific). Dead cells were excluded by Near-IR dead cell staining (Invitrogen). CountBright Absolute Counting Beads (Thermo Scientific) were added to each sample to calculate total cell numbers. Gating strategy is provided in Supplementary Fig. S2e.

### Evaluation of distant metastasis in mouse lungs

Lung metastases burden was quantified on Day 21 by haematoxylin–eosin (HE) staining. Mouse lungs were fixed in 4% paraformaldehyde, embedded in OCT, snap-frozen, and stored at −80 °C. Horizontal 8 µm sections were cut every 1 mm; three levels per lung (0, 1, and 2 mm, starting 200 µm from the apex) were selected for analysis. Sections were stained using a standard Mayer’s haematoxylin–eosin protocol, dehydrated, cleared, and mounted. Bright-field images were captured, and metastasis lesions were quantified on the three predefined levels of each lung.

### Immunofluorescence staining to evaluate spatial distribution of EGFR ligands and vascular structure in 4T1 tumor tissues

Frozen tumor sections were thawed, re-hydrated in PBS, and blocked with Akoya blocking buffer to minimize non-specific binding. Slides were incubated overnight at 4 °C with the following primary antibodies: LYVE-1 (1:100, 50-0443-80, eBioscience), CD31 (1:400, MA3105, Invitrogen), CTGF (1:200, ab6992, Abcam), and TGF-α (1:200, ab9585, Abcam). After PBS washes, sections were labelled for 1 h at room temperature with species-specific secondary antibodies—Alexa Fluor 555 anti-rabbit (1:200, A32731, Invitrogen) and Alexa Fluor 488 anti-hamster (1:1 000, A78963, Invitrogen)—followed by Hoechst counterstaining for nuclear labeling. Fluorescence images were acquired on a Vectra 3 automated imaging system (Akoya Biosciences) at 10× or 20× magnification using the appropriate filter sets for Alexa 488, Alexa 555, and DAPI. Quantitative assessment of signal intensity was performed in QuPath (version 0.5.1). For each tumor, around two regions of interest (ROIs) were acquired. Within each ROI, CD31⁺ vascular areas and LYVE-1⁺ lymphatic areas were segmented based on fluorescence intensity thresholds. Median fluorescence intensities of TGFα and CTGF within the CD31⁺ and LYVE-1⁺ segmented regions were then extracted for subsequent statistical analysis.

### Evaluation of immune cell composition in TDLNs

For T cell profiling, single-cell suspensions were stained with surface markers including VioBright FITC-anti-CD8a (130-120-822, clone REA601, Miltenyi Biotec), PE-Cy7-anti-CD4 (100528, clone RM4-5, BioLegend), BV605-anti-CD25 (102035, clone PC61, BioLegend), and BV650-anti-CD3 (100229, clone 17A2, BioLegend). Alexa Fluor 647-anti-FoxP3 (126407, clone MF-14, BioLegend) was used for intracellular staining following fixation and permeabilization. Intracellular staining was performed according to the manufacturer’s instructions using FoxP3 Fix/Perm buffer set (00-5521-00, Invitrogen). Data were acquired on a Cytoflex cytometer (Beckman Coulter Life Sciences) and analyzed with FlowJo software (version 10.10.0; FlowJo, LLC). Gating strategy is provided in Supplementary Fig. S9.

### TGF-α blockade using Fepixnebart

To investigate the therapeutic potential of TGF-α neutralization in lymphatic metastasis, we utilized Fepixnebart (HY-P990071, MedChemExpress), a humanized IgG4 monoclonal antibody with high affinity for TGF-α and epiregulin. For in vivo treatment, mice were injected orthotopically with 1 × 10⁵ 4T1-EGFR cells into the fourth mammary fat pad. Simultaneously, mice received a single intravenous dose of Fepixnebart (10 mg/kg) or an isotype-matched human IgG4 control antibody (HY-P99003, MedChemExpress). On day 10 post-injection, inguinal (ILN) and axillary (ALN) lymph nodes were harvested, processed into single-cell suspensions, and analyzed by flow cytometry for cytokeratin-positive (CK⁺) tumor cells.

For in vitro invasion assays, 4T1-EGFR cells (1 × 10⁵) were seeded in the upper chambers of Matrigel-coated transwell inserts in serum-free DMEM. The lower chambers were filled with conditioned media from TGF-β1–activated primary lymphatic endothelial cells (svLECs), supplemented with either Fepixnebart (10 μg/mL) or IgG control. After 48 hours, invaded cells were fixed, stained with crystal violet, and quantified in three fields per well across three biological replicates. Statistical comparisons were made using an unpaired two-tailed Student’s t-test.

### Chemotaxis assays

Chemotaxis assays were performed using the µ-Slide Chemotaxis 3D ibiTreat (Ibidi GmbH, Germany). The cells were pre-treated with 10 μg/mL mitomycin in serum-free medium for 1.5 h at 37 °C to inhibit cell proliferation. The slide’s observation area was filled with cells suspended at a density of 3 × 10⁶ cells/ml in 0.8 mg/ml collagen I and polymerized at 37 °C for 30 min. A chemotactic gradient was established by adding media with chemoattractant to one reservoir, while the other reservoir contained only the base medium as a control. The slide was placed in a stage-top incubator set at 37 °C with 5% CO₂ for live-cell imaging. Time-lapse images of cell migration were captured every 30 minutes for a 48 h period using a phase-contrast microscope. Cells were segmented using Cellpose 2.2^42^ via the ImageJ plugin interface. Resulting segmentation masks were analyzed in TrackMate. Cell tracks were exported and subsequently analyzed using the Chemotaxis and Migration Tool (Ibidi, Version 2.0), with parameters such as migration distance, velocity, and directionality calculated to assess chemotactic response.

### Invasion assays

Invasion assays were conducted using 8 μm pore cell culture inserts (Merck-Millipore, Solna, Sweden). 70 ul of growth factor reduced Matrigel at concentration of 300 ng/ml was added to the inserts and allowed to solidify for 1 h in a 37 °C incubator. A total of 100,000 cells, suspended in 300 µl of RPMI medium without FBS, were seeded on top of the Matrigel layer in each insert. The inserts were then placed in separate wells of a 24-well plate, each containing 700 µl of either control medium (RPMI without FBS), conditioned medium, or medium supplemented with growth factors. After 48 hours, cells that had migrated to the lower chamber were stained with crystal violet and imaged using a brightfield microscope. Three randomized fields were captured for each sample, and the pixel area of the stained regions was quantified using ImageJ.

### Wound-healing assays

Wound-healing assays were performed using Culture-Inserts 2 Well (Ibidi GmbH, Germany). Inserts were placed in 24-well plates, and cells were seeded at a density of 50,000 cells per well in each compartment of the inserts in complete RPMI medium with 10% FBS. Cells were allowed to attach and reach confluence over 24 h in a 37 °C incubator with 5% CO₂. After achieving confluence, the inserts were carefully removed to create a defined gap, and the wells were gently washed with PBS to remove detached cells. The medium was then replaced with control medium (RPMI without FBS), conditioned medium, or medium supplemented with growth factors. Images of the wound area were captured at every 30 min using phase-contrast microscope equipped with an on-stage incubator to maintain controlled conditions. The wound area was measured and analyzed using ImageJ software to calculate the percentage of wound closure. Experiments were conducted in triplicate.

### Western Blot analysis

Cell lysates were prepared in ice-cold RIPA buffer supplemented with protease- and phosphatase-inhibitor cocktails according to previous protocol^43^. To recover secreted proteins from culture supernatants, pre-chilled acetone was added, and samples were vortexed and kept at −20 °C for 1 h, then centrifuged at 14,000 × *g* for 10 min. The supernatant was carefully decanted, and residual acetone was allowed to evaporate for 30 min at room temperature before pellets were resuspended in RIPA buffer. Protein concentrations were determined with the BCA assay (Thermo Fisher Scientific, 23227). Equal amounts of protein were separated by SDS-PAGE and transferred to PVDF membranes. Membranes were blocked for 1 h in 3% (w/v) non-fat milk and probed overnight at 4 °C with the following primary antibodies: anti-Akt (pan) (C67E7, #4691S), anti-phospho-Akt (Ser473) (D9E, #4060S), anti-STAT3 (#MAB-1799), anti-phospho-STAT3 (Tyr705) (M9C3, #4113S), anti-ERK1/2 (p44/42 MAPK) (137F5, #4695 P), anti-phospho-ERK1/2 (Thr202/Tyr204) (#4370 P), anti-EGFR (D38B1, #4267), anti-epiregulin (EREG, #Ab233512), anti-HB-EGF (#AF-8239-SP), anti-CTGF (#Ab6992), anti-TGF-α (#Ab9585), and anti-GAPDH (GA1R, MA5-15738-HRP) as loading control. After washing, blots were incubated for 1 h at room temperature with HRP-conjugated goat anti-rabbit IgG (1:5000; Cell Signaling Technology). Signal was visualized using enhanced chemiluminescence (34580, Thermo Scientific). When probing for proteins of similar molecular weight, membranes were stripped with stripping buffer (R-03722-D50, Advansta), washed, and re-blocked followed by re-incubation with the next primary antibody.

### Quantitative real-rime RT–PCR analysis

The extraction of total RNA was done by using the RNeasy mini kit (Qiagen) according to the manufacturer’s instructions. cDNA synthesis was done by QuantiTect Reverse Transcription kit (Qiagen) using an amount of 1 µg of total RNA. For qPCR (quantitative realtime PCR) analysis, cDNA mixture was used in an amount of 5 ng for PCR amplification by QuantiTect SYBR Green PCR Kit (Qiagen) with validated QuantiTect primers (Qiagen). The following genes were analyzed: Mouse EGFR (forward 5′-GGACTGTGTCTCCTGCCAGAAT-3′ and reverse 5′-GGCAGACATTCTGGATGGCACT-3′). The PCR was carried out as follows: 3 min at 95 °C followed by 35 cycles of 3 s at 95 °C, 20 s at 55 °C and 2 s extension step at 72 °C in Biorad PCR system.

### Single-cell RNAseq data analysis

Raw gene-count matrices were downloaded from the Gene Expression Omnibus (Access number: GSE167036). Counts and the corresponding metadata—containing per-cell quality-control metrics, UMAP embeddings, clustering assignments, and author-defined cell-type labels—were combined and imported into Seurat (v4.4.0). Because the original study had already performed filtering and dimensionality reduction, we retained those values and did not repeat the upstream QC pipeline. Genes of interest were displayed with standard Seurat plotting functions (FeaturePlot, VlnPlot) to compare expression across clusters and annotated cell types.

### Bulk RNAseq data analysis

Raw sequencing data were retrieved from the GEO (Access number: GSE241881) and processed as previously described^43^. Log-transformed counts per million (log-CPM) were used for visualization. Differential expressions were assessed with limma-voom (limma v3.56.2); genes displayed in the figures met a false-discovery-rate threshold of <0.05.

### GOBO database analysis

Gene expression and survival analyses were performed using the GOBO (Gene expression-based Outcome for Breast cancer Online) tool (version 1.0.3, available at https://co.bmc.lu.se/gobo). Analyses were conducted using the “Gene Set Analysis – Tumors” and “Sample Prediction” modules to assess the association of *TGFA* and CTGF with clinical subtypes, survival outcomes.

### Public gene expression datasets analysis

Gene expression and survival analyses were performed using the GOBO (Gene expression-based Outcome for Breast Cancer Online) tool (version 1.0.3; https://co.bmc.lu.se/gobo), which integrates multiple breast cancer microarray datasets. Analyses were conducted using the “Gene Set Analysis – Tumors” and “Sample Prediction” modules to assess the association of TGFA and CTGF expression with intrinsic molecular subtypes and clinical outcomes. Independent validation was performed using the METABRIC (Molecular Taxonomy of Breast Cancer International Consortium) cohort. Normalized gene expression data and clinical annotations were obtained from cBioPortal for Cancer Genomics (https://www.cbioportal.org). Tumors were stratified according to PAM50 intrinsic subtypes (Basal-like, HER2-enriched, Luminal A, Luminal B, Normal-like, and Unclassified).

### Statistical analysis

Investigators were not blinded during experimental procedures, but outcome quantification and statistical analyses were performed using coded datasets. Statistical analyses were performed using GraphPad Prism (version 10) and R (version 4.3.2). Results are presented as mean ± standard error of the mean (SEM) unless otherwise indicated. Sample sizes were estimated based on pilot experiments. Power calculations were performed assuming an α level of 0.05 and a statistical power of 80% to detect biologically relevant differences between groups. For comparisons between two groups, unpaired two-tailed Student’s *t*-tests were used. For comparisons involving more than two groups, one-way or two-way analysis of variance (ANOVA) followed by Tukey’s multiple comparisons post hoc test was applied as appropriate. P-values less than 0.05 were considered statistically significant. Data represent SEM with three independent experiments in triplicate.

For cell migration, invasion, and wound-healing assays, quantification was based on pixel intensity or wound closure area from at least three independent biological replicates, each with technical triplicates unless otherwise stated. For chemotaxis assays, directional migration was evaluated using the Forward Migration Index (FMI), and statistical significance of vectorial migration was assessed by the Rayleigh test. Flow cytometry-based cell population analyses and tumor burden quantifications were normalized using counting beads and analyzed across at least three mice per group and time point. Statistical tests used are reported in the figure legends.

## Supplementary information

41523_2026_941_MOESM1_ESM

41523_2026_941_MOESM2_ESM

41523_2026_941_MOESM3_ESM

## Data Availability

The single-cell RNA-sequencing dataset analyzed in this study is publicly available at the NCBI Gene Expression Omnibus (GEO) under accession number **GSE167036**. The bulk RNA-sequencing dataset used to assess TGF-β1-induced gene expression in lymphatic endothelial cells is available under GEO accession number **GSE241881**. All other relevant datasets generated and analyzed during the current study are available from the corresponding author upon reasonable request. Code for the single-cell transcriptomic analysis presented in this study is publicly accessible via GitHub: https://github.com/WenyangS/Endo_scRNAseq.
