# Supplementary material for: TGF-α/EGFR-mediated lymphatic metastasis reveals a repositionable therapeutic target in breast cancer
Source: NPJ Breast Cancer. 2026 Apr 3;12:52. doi: 10.1038/s41523-026-00941-0 (PMC13057184; doi:10.1038/s41523-026-00941-0)
Supplement: Supplementary file 1 — 41523_2026_941_MOESM1_ESM [file 41523_2026_941_MOESM1_ESM.pdf]

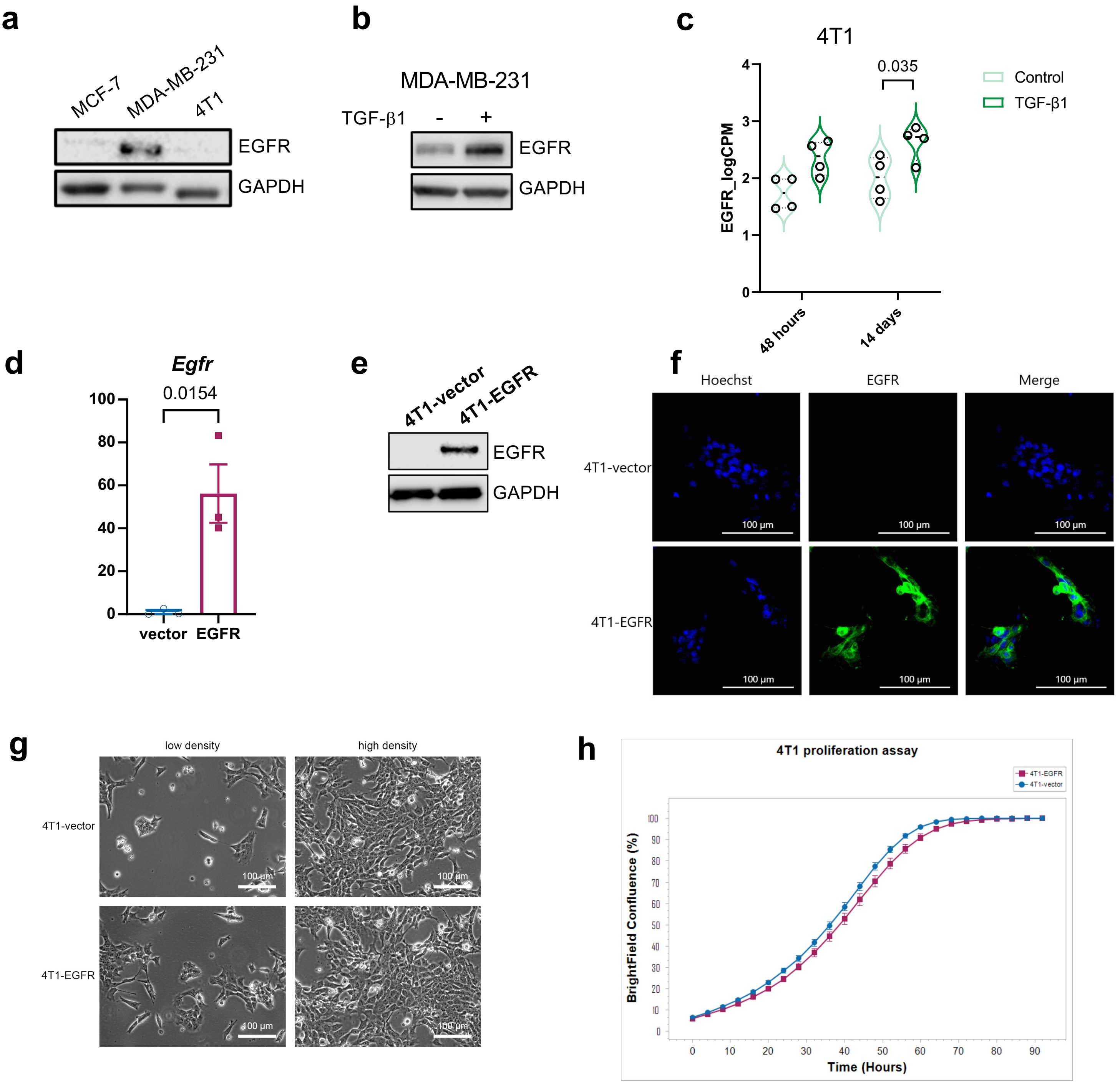

Supplementary Figure S1. TGF-β1 upregulates EGFR expression in breast cancer cells, but EGFR overexpression does not alter tumor proliferation. a Western Blot analysis of EGFR expression in MCF-7, MDA-MB-231 and 4T1 cell lines. **b** Western blot results showing upregulation of EGFR expression in MDA-MB-231 cells after TGF-β1 treatment. **c** Violin plot showing upregulation of *EGFR* mRNA levels in 4T1 cells treated by TGF-β1 for 48 hours or 14 days. Data was derived from unpublished bulk RNA sequencing. Y-axis indicates log counts per million (log-CPM). **d** Results from qPCR analysis of EGFR mRNA levels in 4T1 cells engineered to overexpress EGFR (4T1-EGFR) compared to control cells (4T1-vector). **e** Western Blot analysis of stable EGFR overexpression in 4T1 cells compared to control cells (vector). **f** Immunofluorescence images showing staining of EGFR (green) in 4T1-vector and 4T1-EGFR cells. Nucleus is stained with Hoechst (Blue). **g** Representative brightfield images of 4T1-vector and 4T1-EGFR cells under low (left) and high (right) density. **h** Real-time proliferation assay using IncuCyte live-cell imaging. Cell growth (Confluency measured from phase-contrast images) of 4T1-vector and 4T1-EGFR cells were monitored and compared over time (96 hours).

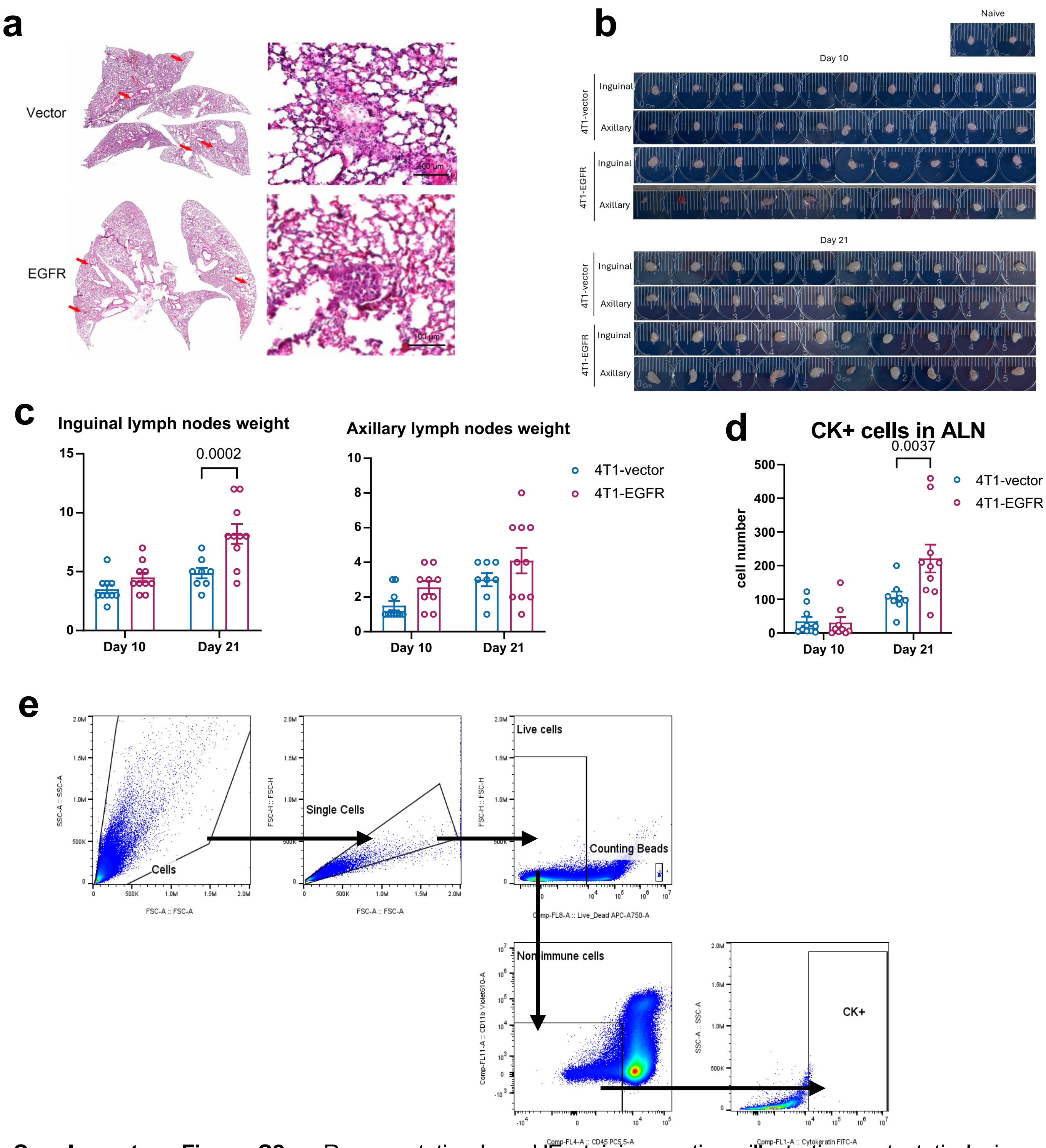

Supplementary Figure S2. a Representative lung HE staining sections illustrating metastatic lesions. Lung metastatic burden was quantified on day 21 using H&E staining. OCT-embedded lungs were serially sectioned at 1,000 μm intervals, and three non-adjacent sections per mouse were analyzed to minimize overestimation of metastatic foci. **b** Representative images of inguinal and axillary lymph nodes harvested at different time points after tumor implantation. The surrounding connective tissue was removed prior to imaging. The naïve lymph node (upper right) was collected from the contralateral inguinal region of a non-injected control mouse. **c** Weights of tumor-draining lymph nodes were measured using a microbalance. Statistical significance among groups was assessed using one-way ANOVA. Data are presented as mean ± SEM. **d** Cytokeratin-positive (CK^{+}) tumor cells in axillary (ALN) lymph nodes were quantified by flow cytometry at indicated time points. Statistical analysis was performed using one-way ANOVA. Data are presented as mean ± SEM. **e** Gating strategy for tumor cell quantification in draining lymph nodes. Immune cells were excluded by gating on $CD45^{-}CD11b^{-}$ cells to avoid signal contamination from cytokeratin uptake by antigen-presenting cells. Tumor cells were identified as pan-cytokeratin^{+} (CK+) within the $CD45^{-}CD11b^{-}$ population.

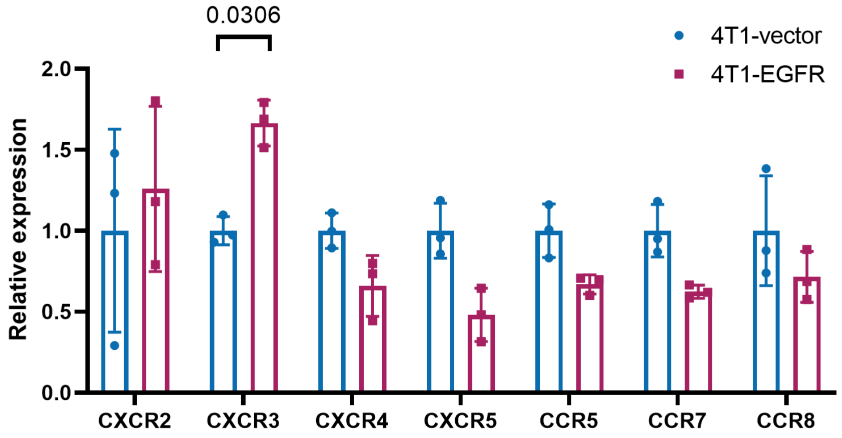

Supplementary Figure S3. Effect of EGFR overexpression on the expression of cytokine receptors. Results from qPCR analysis of lymph node–associated chemokine receptors in 4T1-EGFR and 4T1-vector cells. Receptors analyzed include CXCR2, CXCR3, CXCR4, CXCR5, CCR7, CCR8. Gene expression levels were normalized to housekeeping gene GAPDH and are shown relative to 4T1-vector cells. Data represent mean ± SEM. Statistical analysis was performed using unpaired two-tailed Welch's t-tests for each gene, followed by correction for multiple comparisons using the two-stage step-up false discovery rate procedure of Benjamini, Krieger and Yekutieli. Adjusted q-values < 0.05 were considered statistically significant and are indicated in the graph.

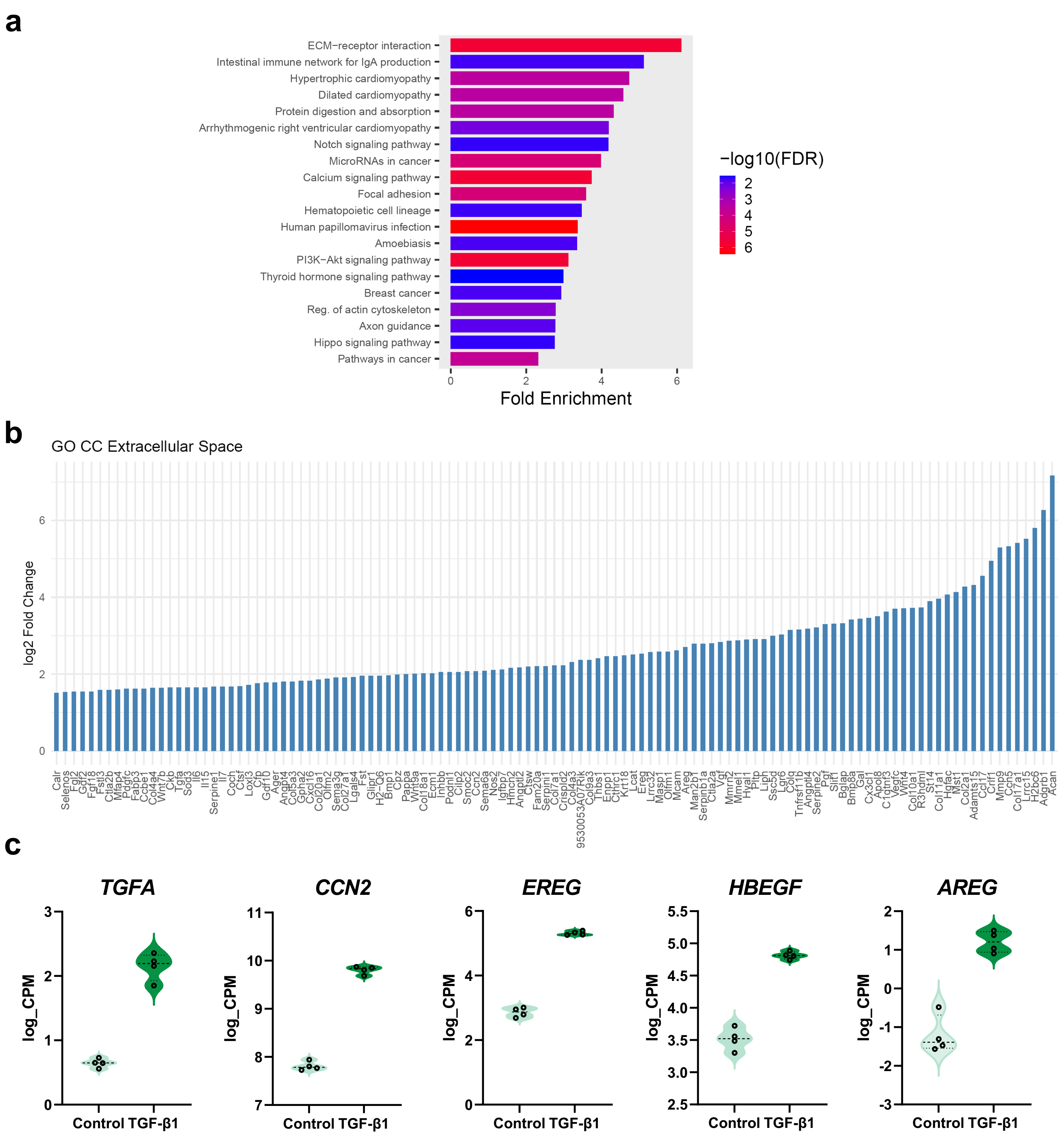

Supplementary Figure S4. Identification of EGFR ligands in the secretome of TGF-β1-activated lymphatic endothelial cells. a Gene enrichment analysis of KEGG pathways was performed to compare transcriptomic changes before and after TGF-β1 treatment. The top 20 enriched pathways are shown, ranked by fold enrichment. **b** Genes encoding proteins in extracellular space (GO cellular component (GO:CC)) that were upregulated upon TGF-β1 treatment in svLEC cells. **c** Violin plots show the upregulation of EGFR ligands at the mRNA level in svLEC cells before (control, n = 4) and after (TGF-β1 n = 4) TGF-β1 treatment. Y-axis indicates log counts per million (log-CPM). Differential expression analysis was performed, and all shown genes exhibited statistically significant increases with FDR < 0.05.

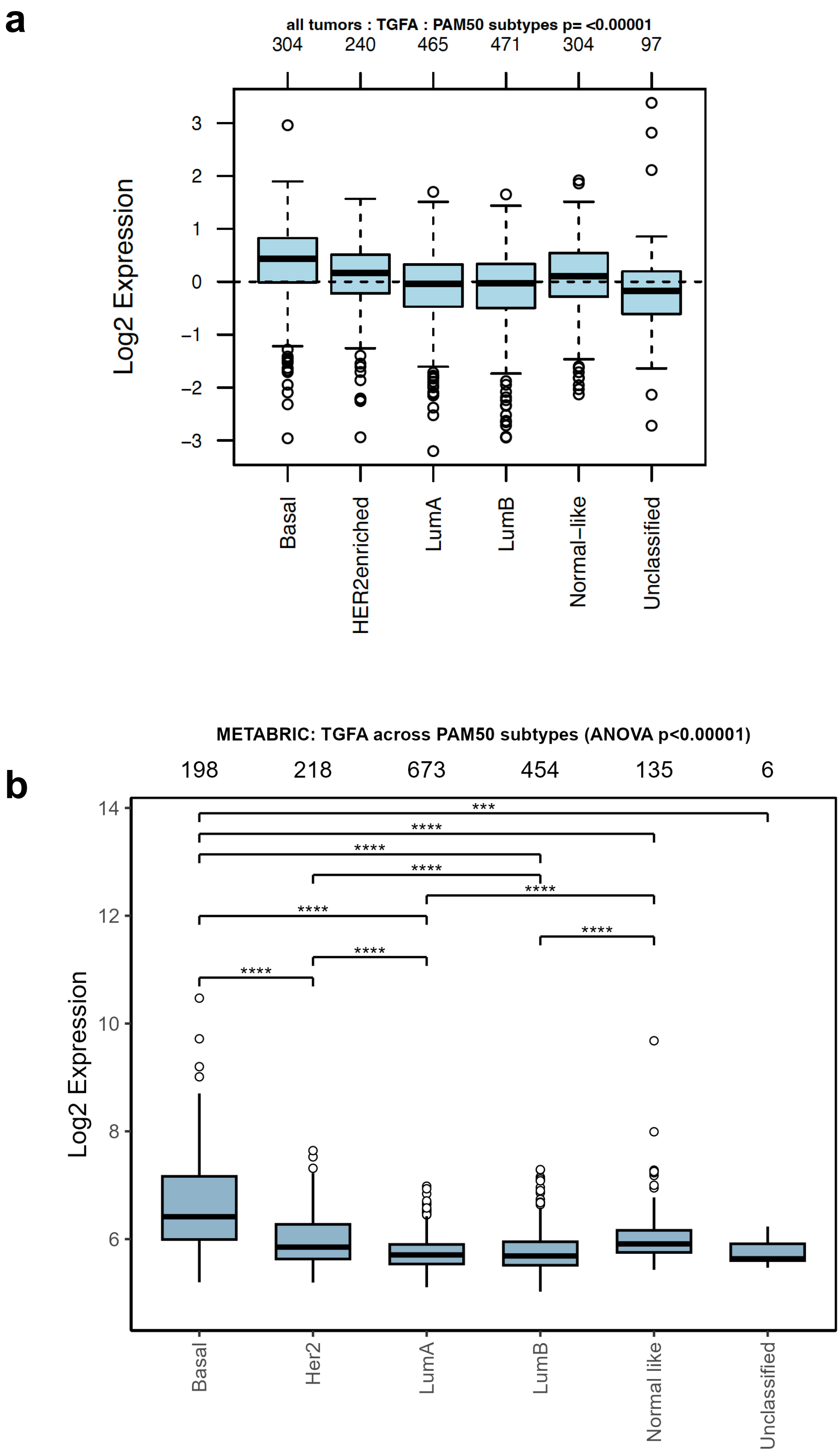

Supplementary Figure S5. Expression of TGFA in different breast cancer subtypes. Log2-transformed TGFA expression levels across PAM50 breast cancer subtypes in the (a) GOBO database and (b) METABRIC cohort. Breast cancer intrinsic subtypes include Basal-like, HER2-enriched, Luminal A, Luminal B, Normal-like, and Unclassified tumors. Sample sizes for each subtype are indicated above the plots. Overall differences among subtypes were assessed by one-way ANOVA. Multiple comparisons in panel (b) were performed using Tukey's honestly significant difference post hoc test. Significant differences are indicated by asterisks (*p < 0.05, **p < 0.01, ***p < 0.001, ****p < 0.0001).

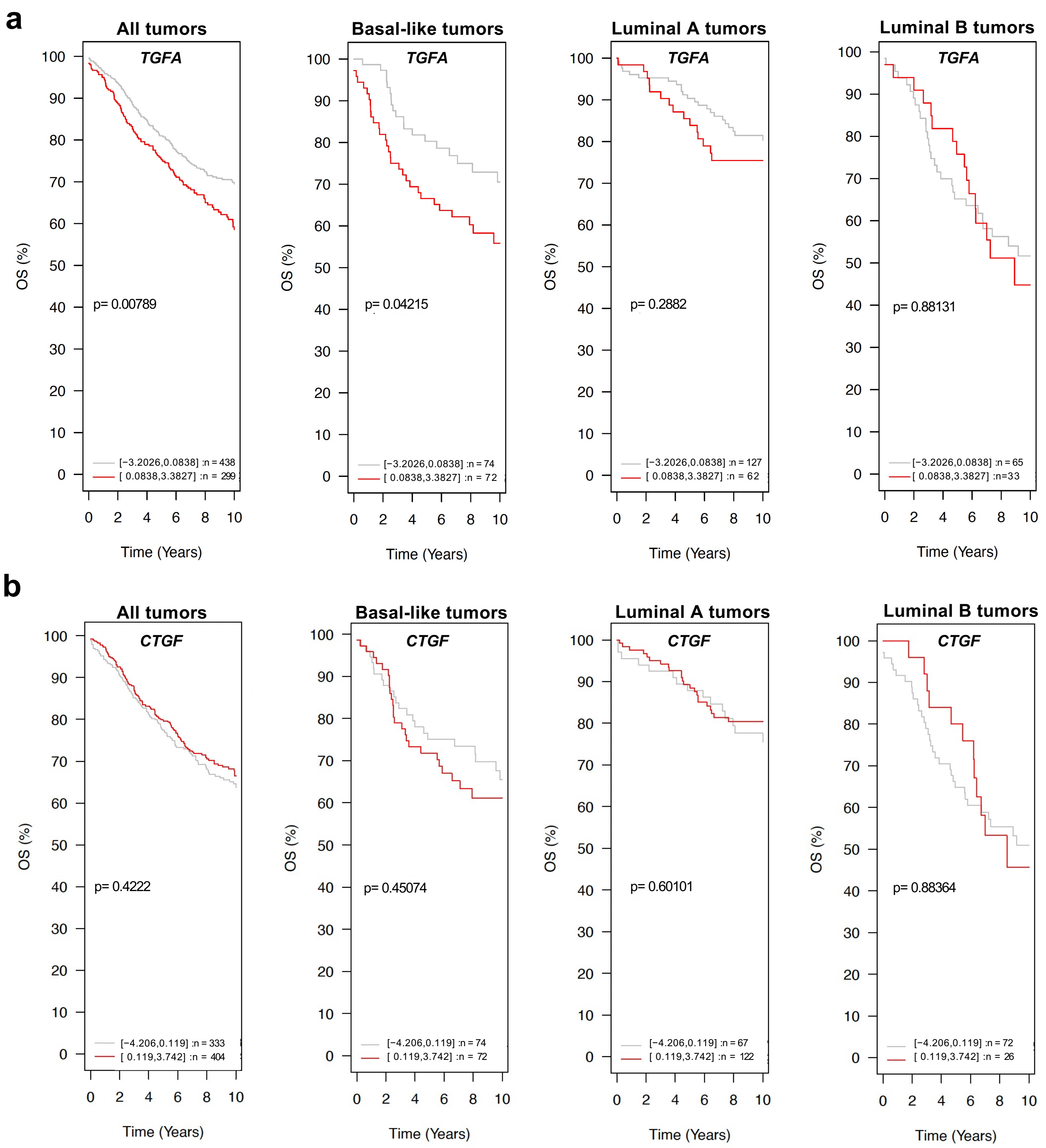

Supplementary Figure S6. High *TGFA* expression correlates with poor survival in breast cancer patients. Kaplan–Meier survival analysis of breast cancer patients stratified by tumor expression levels of *TGFA* (**a**) and *CTGF* (**b**) using the GOBO (Gene expression-based Outcome for Breast cancer Online) database (Lund University, Sweden). Survival curves are shown for all breast cancer subtypes (All tumors), as well as for major intrinsic subtypes: basal-like, luminal A, and luminal B tumors. Patients were stratified into high (red) and low (grey) expression groups based on tumor gene expression levels (values within brackets). High TGFA expression is associated with significantly worse prognosis in all tumors, specifically in basal-like tumors, whereas CTGF expression shows no prognostic impact. n = number of patient samples per group.

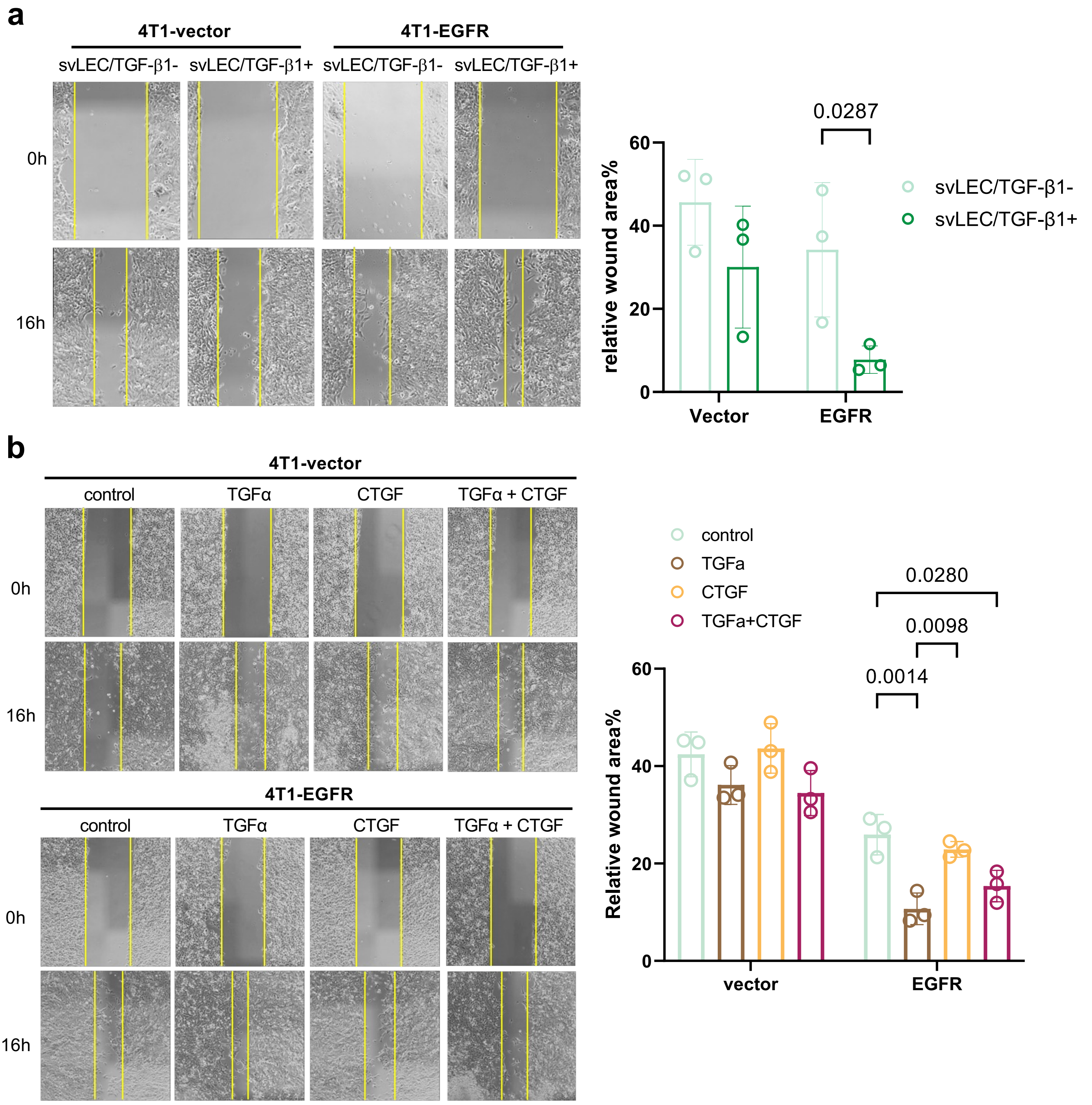

Supplementary Figure S7. Migration of 4T1-EGFR cells toward lymphatic signals. a-b Results from wound healing assays testing the capacity of 4T1-vector and 4T1-EGFR cells to migrate toward conditioned medium (CondM) from svLECs, with or without TGF-β1 stimulation (**a**), and the EGFR ligands TGFa and CTGF, alone or in combination (**b**). Wound closure was monitored by live-cell imaging every 15 minutes. The endpoint wound closure is shown as a bar graph. Statistical analysis was performed using two-way ANOVA.

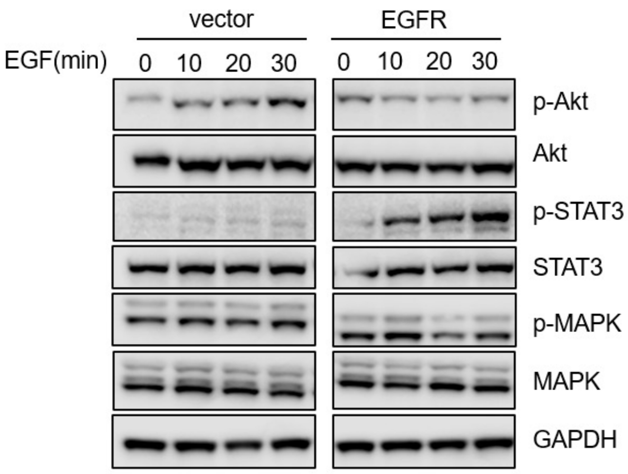

Supplementary Figure S8. Western Blot analysis of stable EGFR overexpression in 4T1 cells compared to vector control cells.

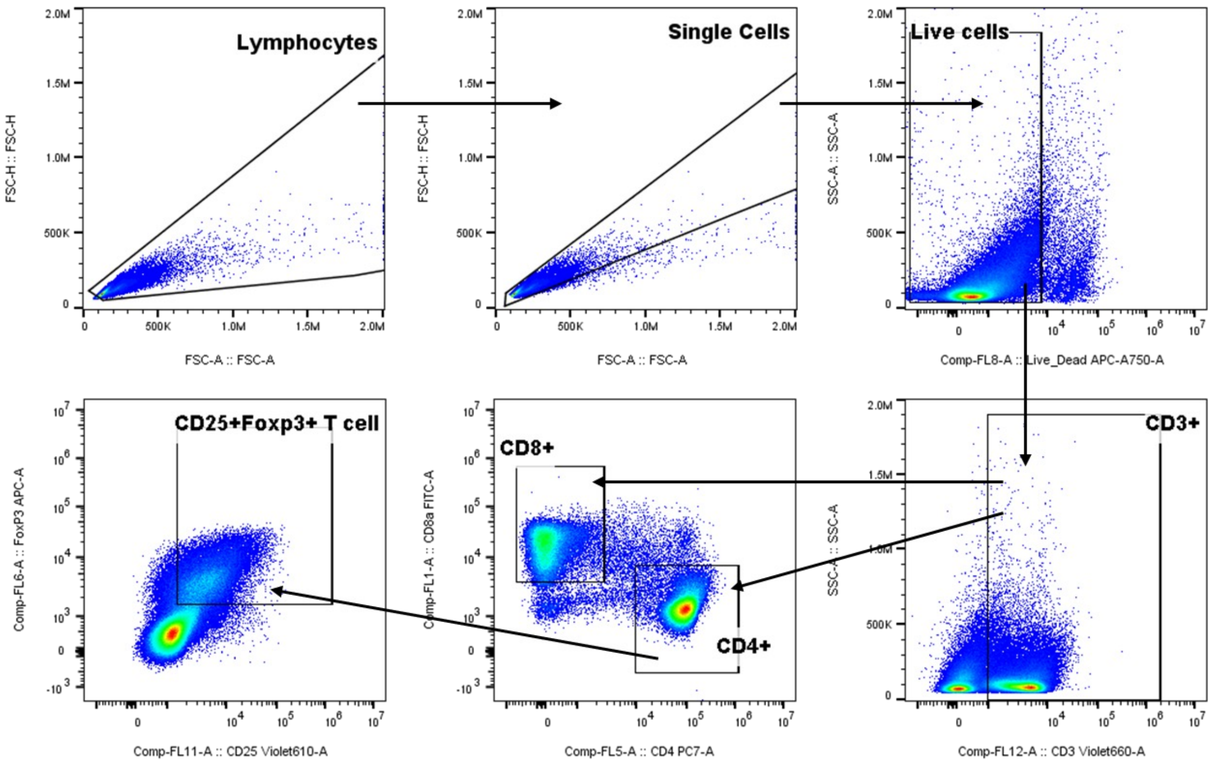

Supplementary Figure S9. Gating strategy for T cell profiling in tumor-draining lymph nodes. Flow cytometry gating strategy used to identify $CD3^+$ T cells, $CD4^+$ and $CD8^+$ subsets, and regulatory T cells (Tregs) in single-cell suspensions from lymph nodes. Live lymph node cells were first gated based on forward and side scatter, followed by singlet discrimination. $CD3^+$ T cells were identified and subsequently gated for $CD4^+$ and $CD8a^+$ expression. Regulatory T cells (Tregs) were defined as $CD4^+CD25^+FoxP3^+$ cells after intracellular staining using the FoxP3 Fix/Perm buffer set. Data acquisition was performed using a CytoFLEX flow cytometer and analyzed with FlowJo software.

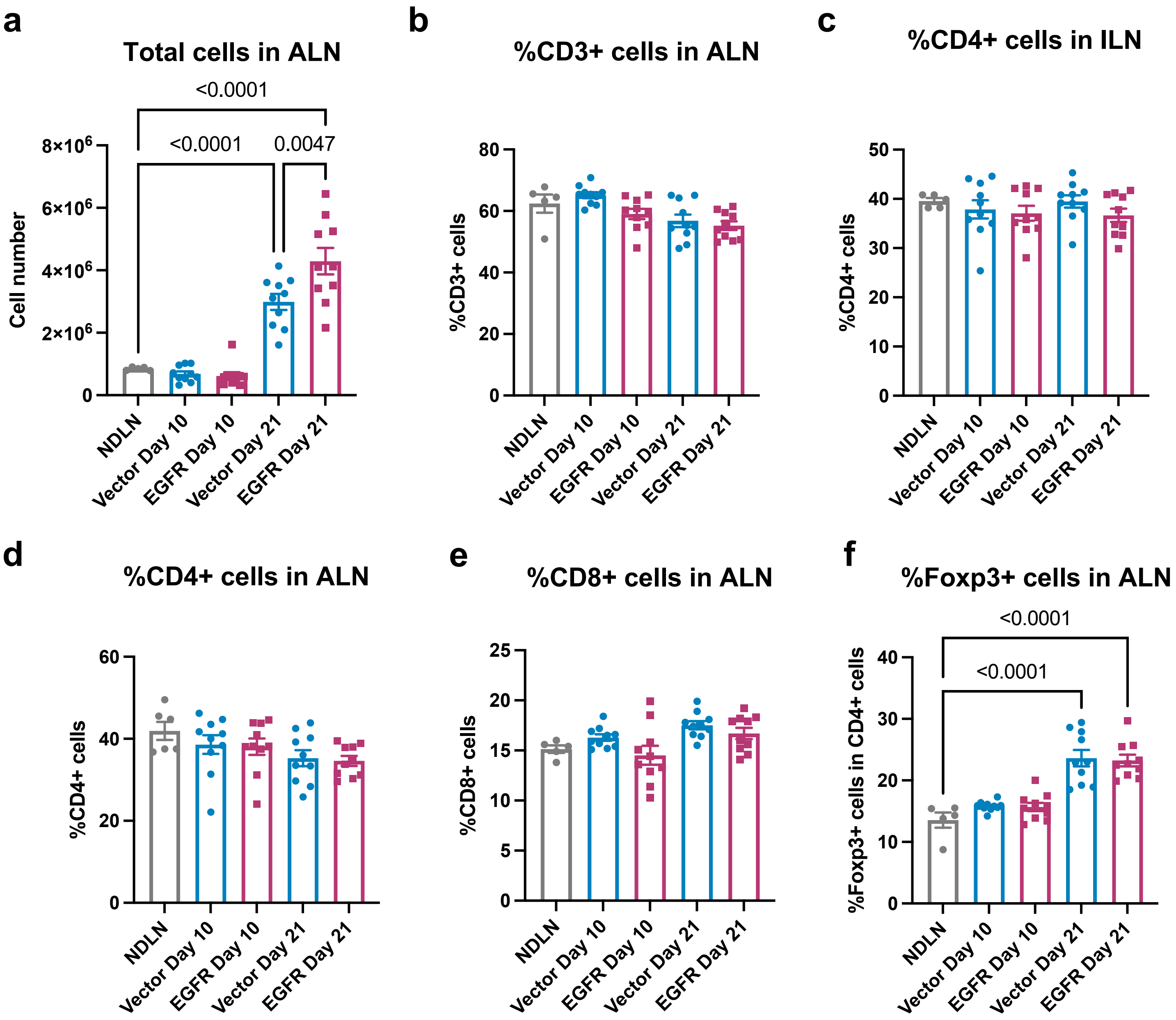

Supplementary Figure S10. Cellularity and an immunoregulatory compositional shift in tumor-draining lymph nodes. a Total number of cells in axillary lymph nodes (ALN) compared to non-draining lymph node (NDLN) at day 10, and day 21 following orthotopic implantation of 4T1-vector or 4T1-EGFR cells. **b-f** Percentage of $CD4^+$ T cells in ILN compared to NDLN (**c**), percentage of $CD3^+$ T cells (**b**), $CD4^+$ T cells (**d**), CD8+ T cells (**e**) and $Foxp3^+$ regulatory T cells (**f**) in ALN compared to NDLN at day 10, and day 21. Each dot represents one lymph node; group means ± SEM shown. Statistical analysis was performed by two-way ANOVA with post hoc test.
