# Supplementary material for: TGF-α/EGFR-mediated lymphatic metastasis reveals a repositionable therapeutic target in breast cancer
Source: NPJ Breast Cancer. 2026 Apr 3;12:52. doi: 10.1038/s41523-026-00941-0 (PMC13057184; doi:10.1038/s41523-026-00941-0)

Figure 2b

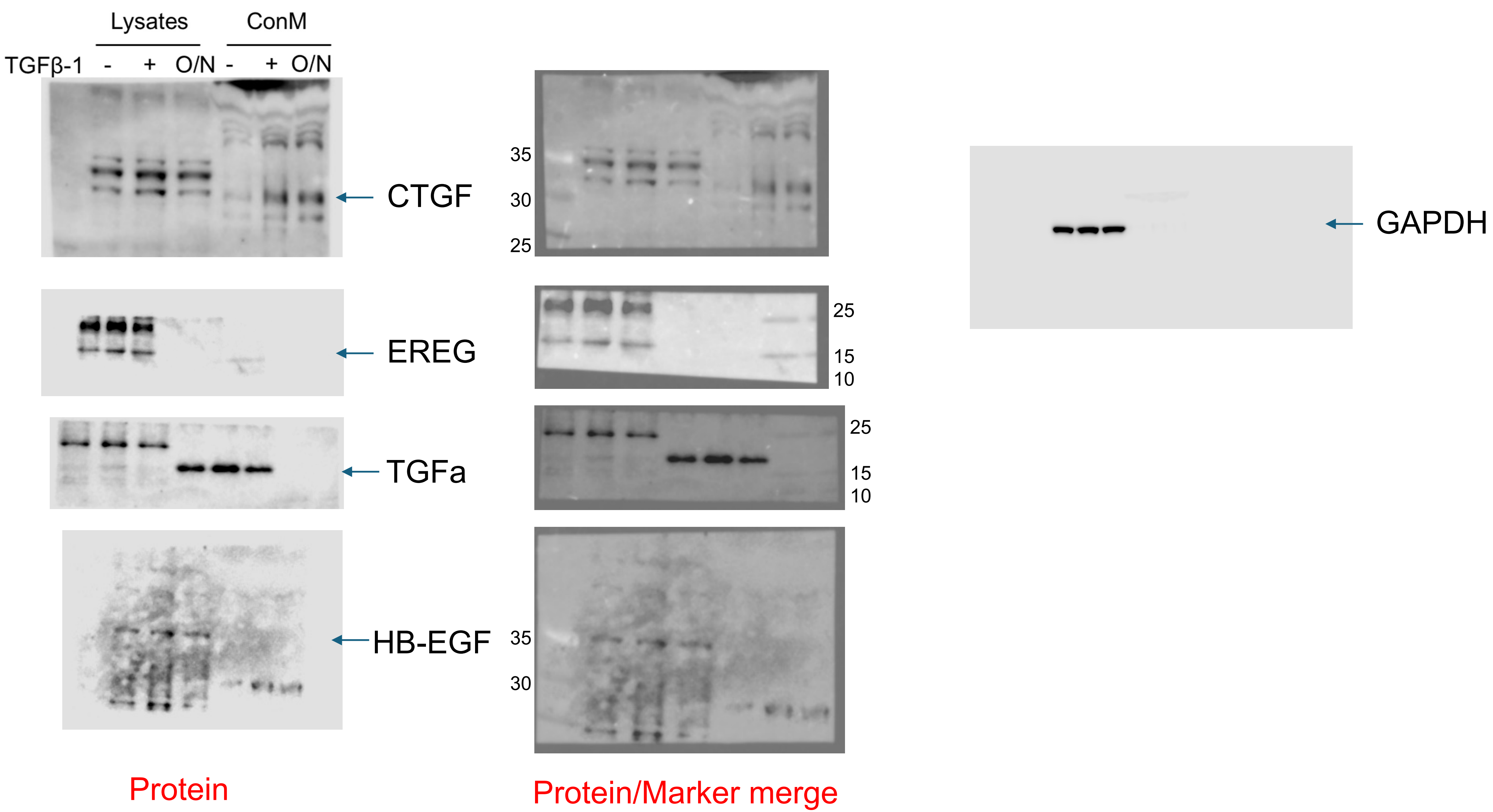

Lysates
ConM
TGFβ-1 - + O/N - + O/N
CTGF
EREG
TGFa
HB-EGF
GAPDH
35
30
25
25
15
10
25
15
10
35
30
Protein
Protein/Marker merge

Figure 4a-c p-Akt/Akt

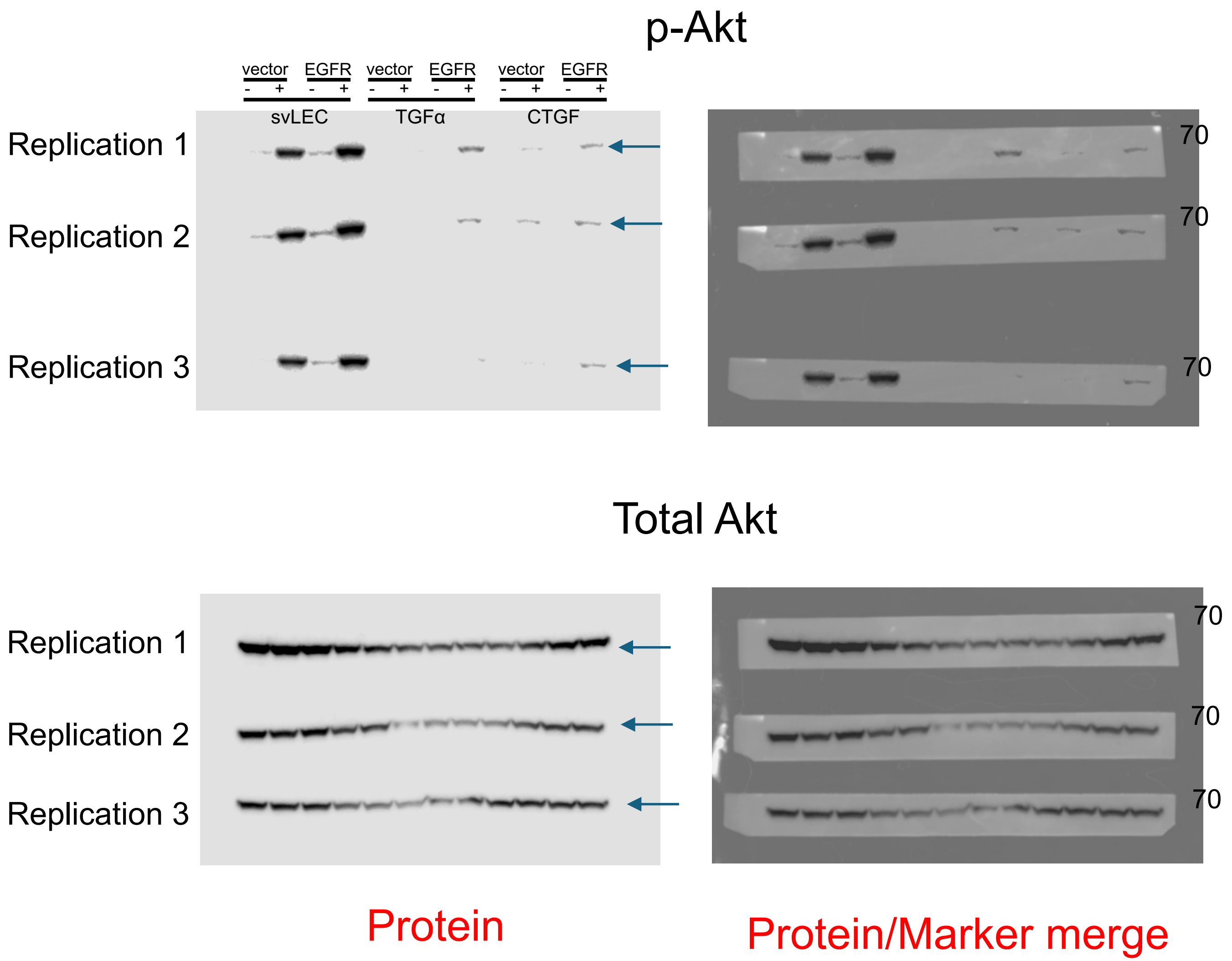

Figure 4a-c p-MAPK/MAPK

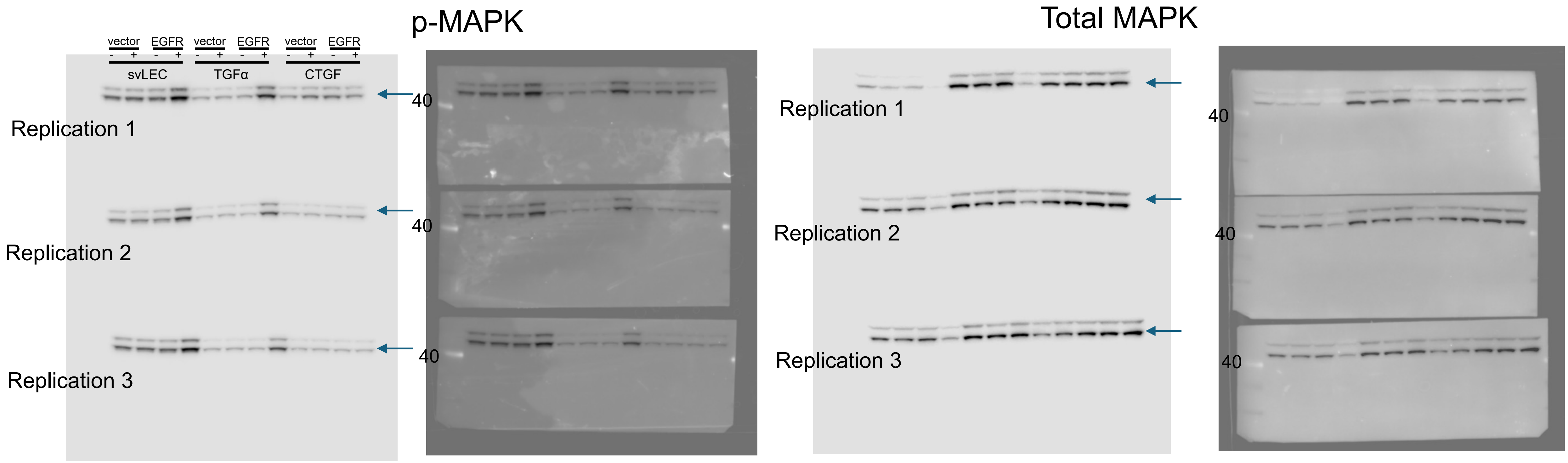

Protein

Protein/Marker merge

Figure 4a-c p-STAT3/STAT3

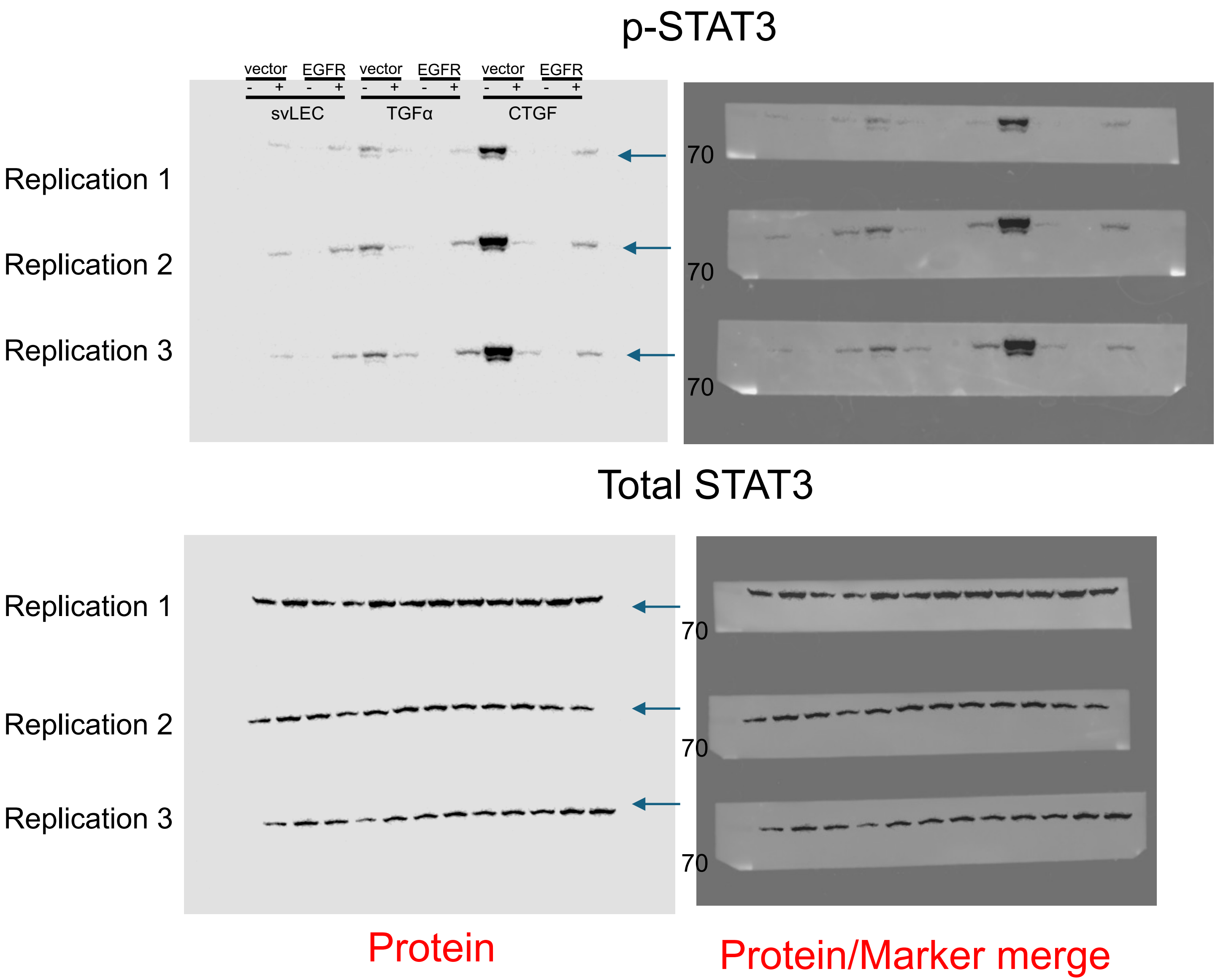

Figure 4a-c GAPDH

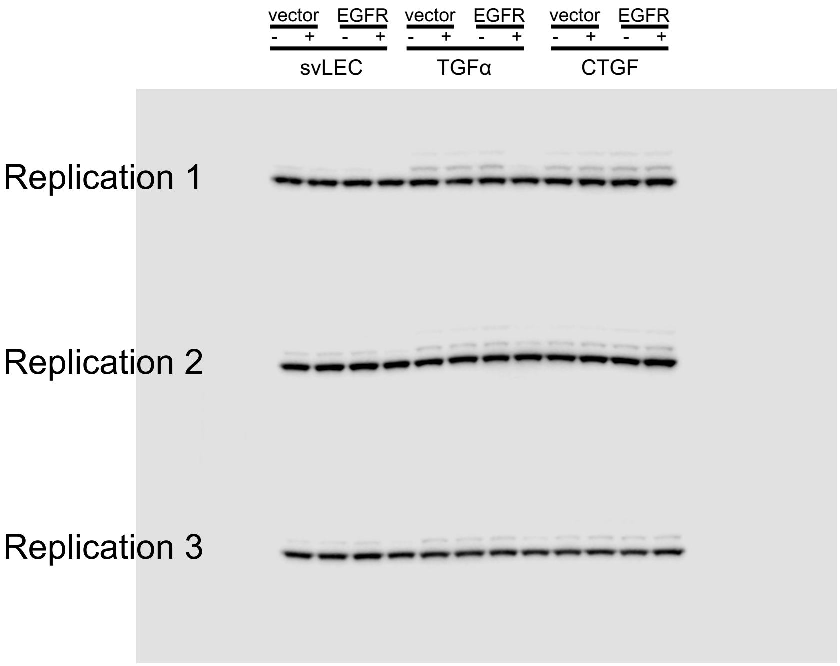

Figure S1 a

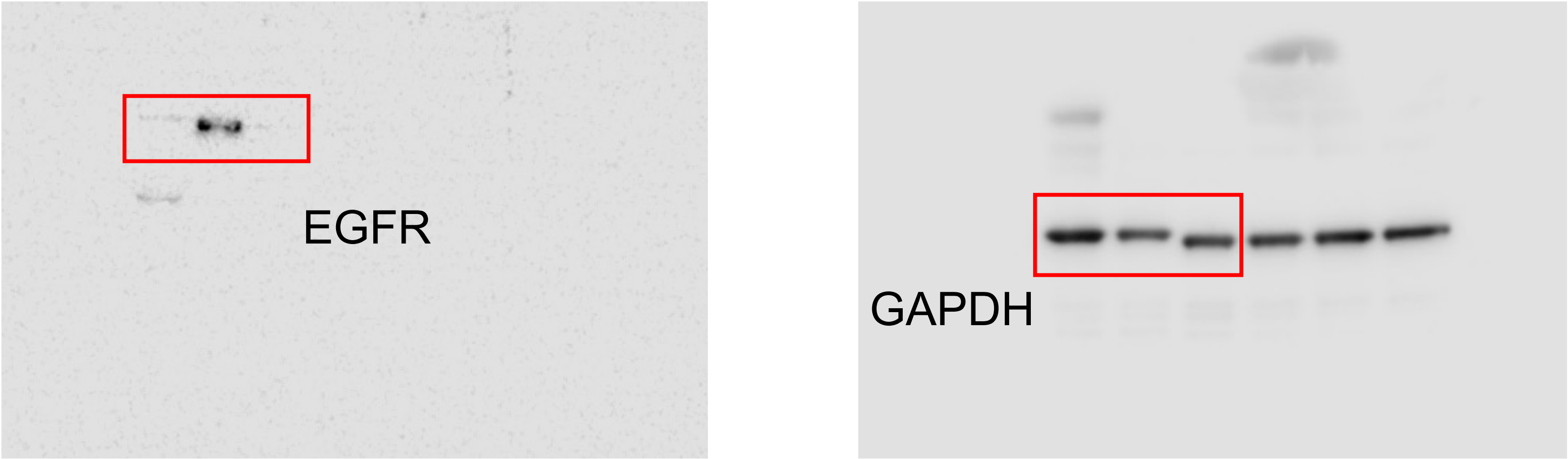

Figure S1 b

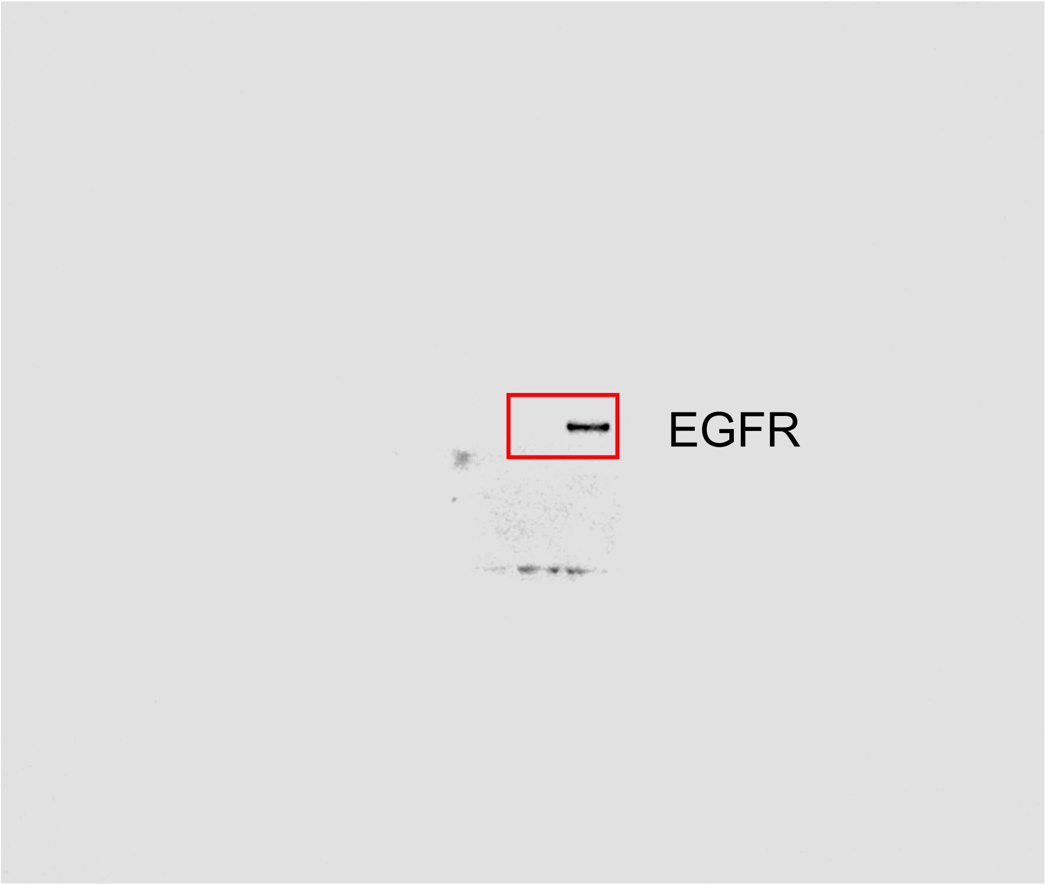

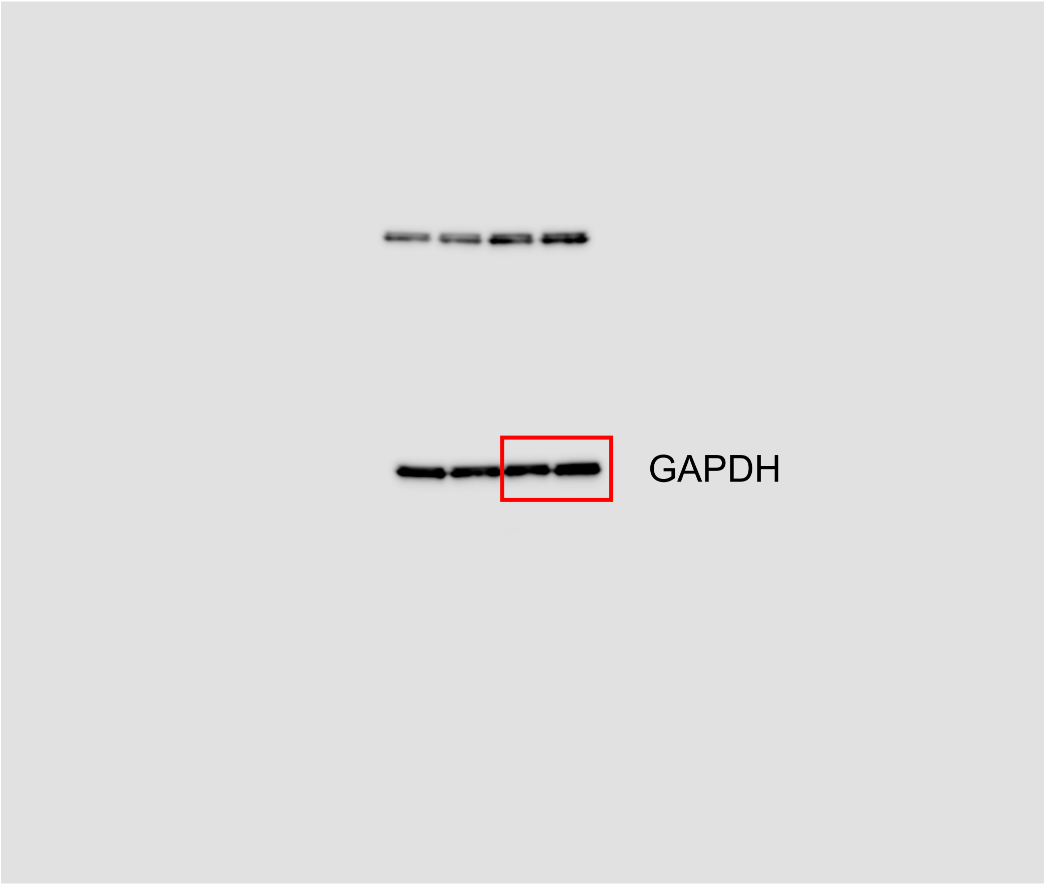

Figure S1 e

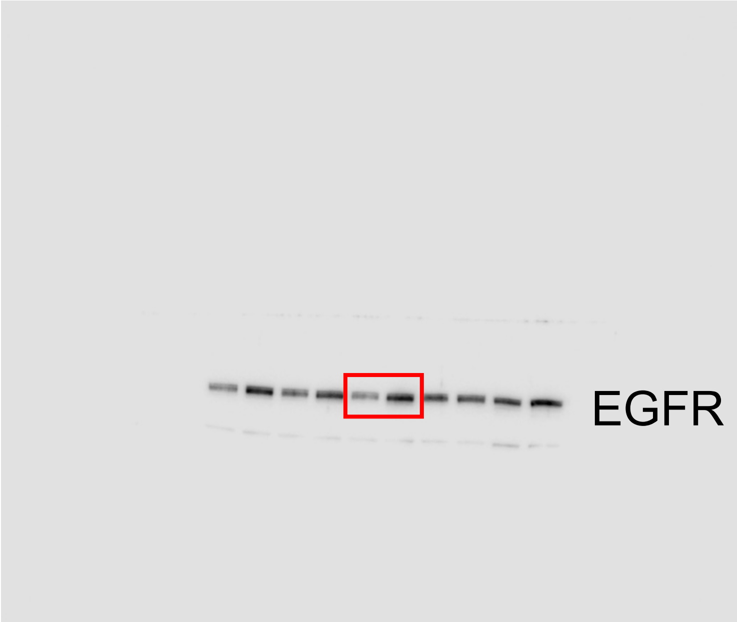

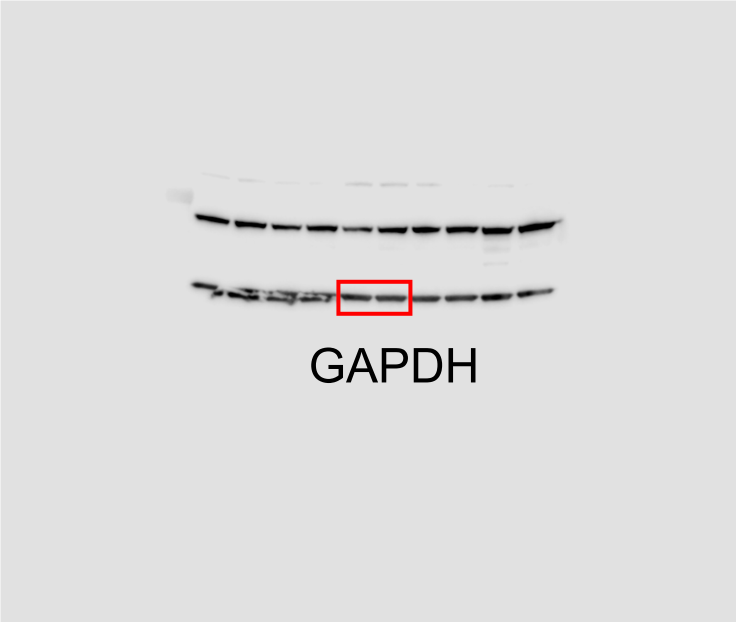

Supplement: Supplementary file 3 — 41523_2026_941_MOESM3_ESM [file 41523_2026_941_MOESM3_ESM.pdf]
